# Adult monozygotic twins discordant for intra-uterine growth have indistinguishable genome-wide DNA methylation profiles

**DOI:** 10.1186/gb-2013-14-5-r44

**Published:** 2013-05-26

**Authors:** Nicole YP Souren, Pavlo Lutsik, Gilles Gasparoni, Sascha Tierling, Jasmin Gries, Matthias Riemenschneider, Jean-Pierre Fryns, Catherine Derom, Maurice P Zeegers, Jörn Walter

**Affiliations:** 1 Laboratory of EpiGenetics, FR 8.3 Life Sciences, Saarland University, Saarbrücken, 66123, Saarland, Germany; 2 Department of Genetics and Cell Biology, Maastricht University, Maastricht, 6200 MD, Limburg, The Netherlands; 3 Nutrition and Toxicology Research Institute Maastricht (NUTRIM), Maastricht University, Maastricht, 6200 MD, Limburg, The Netherlands; 4 Department of Psychiatry and Psychotherapy, Saarland University Hospital, Homburg, 66424, Saarland, Germany; 5 Department of Human Genetics, University Hospital Gasthuisberg, Katholieke Universiteit Leuven, Leuven, B-3000, Vlaams-Brabant, Belgium; 6 Unit of Urologic and Genetic Epidemiology, Department of Public Health and Epidemiology, University of Birmingham, Birmingham, B15 2TT, West Midlands, UK

## Abstract

**Background:**

Low birth weight is associated with an increased adult metabolic disease risk. It is widely discussed that poor intra-uterine conditions could induce long-lasting epigenetic modifications, leading to systemic changes in regulation of metabolic genes. To address this, we acquire genome-wide DNA methylation profiles from saliva DNA in a unique cohort of 17 monozygotic monochorionic female twins very discordant for birth weight. We examine if adverse prenatal growth conditions experienced by the smaller co-twins lead to long-lasting DNA methylation changes.

**Results:**

Overall, co-twins show very similar genome-wide DNA methylation profiles. Since observed differences are almost exclusively caused by variable cellular composition, an original marker-based adjustment strategy was developed to eliminate such variation at affected CpGs. Among adjusted and unchanged CpGs 3,153 are differentially methylated between the heavy and light co-twins at nominal significance, of which 45 show sensible absolute mean β-value differences. Deep bisulfite sequencing of eight such loci reveals that differences remain in the range of technical variation, arguing against a reproducible biological effect. Analysis of methylation in repetitive elements using methylation-dependent primer extension assays also indicates no significant intra-pair differences.

**Conclusions:**

Severe intra-uterine growth differences observed within these monozygotic twins are not associated with long-lasting DNA methylation differences in cells composing saliva, detectable with up-to-date technologies. Additionally, our results indicate that uneven cell type composition can lead to spurious results and should be addressed in epigenomic studies.

## Background

Both observational human and experimental animal studies have confirmed that low birth weight is associated with an increased risk of metabolic diseases, like type 2 diabetes (T2D) [1-3]. Although genetic factors are likely to contribute [4,5], studies assessing the association between low birth weight and T2D precursors in monozygotic (MZ) twins showed that the twin who was lighter at birth had a more adverse metabolic profile in adulthood compared to its genetically identical co-twin, who was heavier at birth [6-10]. This suggests that the association between low birth weight and increased T2D risk is at least partly independent of genetic factors.

One of the possible molecular mechanisms explaining this non-genetic association suggests that poor prenatal conditions induce epigenetic modifications [11]. These epigenetic modifications are believed to cause a 'thrifty' metabolic state, which is beneficial for survival under circumstances of insufficient nutrient supply, but unfavorable when nutrient supply is abundant in postnatal life. An important epigenetic phenomenon is DNA methylation that almost exclusively occurs at cytosines within CpG dinucleotides and correlates with transcriptional repression, while loss of methylation can result in transcriptional activation [12].

The notion that poor intra-uterine conditions cause epigenetic modifications during prenatal development is supported by data from animal studies, where dietary restriction or surgical interventions are used to induce fetal growth restriction, resulting in epigenetic modifications on metabolic disease-related genes (reviewed by [13]). The number of studies assessing the relation between an adverse fetal environment and epigenetic alterations in humans is gradually growing as well. For instance, humans who were periconceptionally exposed to famine during the Dutch Hunger Winter (1944 to 1945) were reported to show significant methylation differences at several imprinted and non-imprinted genes in comparison to their unexposed siblings in peripheral blood cells [14,15]. A genome-wide DNA methylation study performed on CD34+ hematopoietic stem cells from cord blood of five intra-uterine growth restricted (IUGR) neonates and five gestational age and gender-matched controls [16] identified among others significant methylation differences at the *HNF4A *gene, which is involved in monogenic diabetes.

However, epigenetic association studies using population- or family-based designs suffer from confounding caused by DNA sequence variation. Specifically, since birth weight is partly controlled by genetic factors [17], a study with genetically unmatched cases and controls cannot dissect whether a small size at birth is due to a poor prenatal environment or genetic predisposition. On the other hand, epigenetic variation is often a result of genetic variation - for example, allele specific methylation where the methylation pattern of a DNA molecule is determined by a *cis- *or *trans-*acting genetic variant [18]. Since MZ twins originate from one zygote, they are almost absolutely genetically identical, which makes them ideal to search for epigenetic phenomena associated with phenotypic discordancy. In addition, MZ twins are matched for gender, (gestational) age, maternal factors (for example, parity, age) and a broad range of environmental factors.

Depending on whether the embryo splits during an early or later developmental stage, MZ twins can be dichorionic (DC) or monochorionic (MC), respectively. MZ DC twins have two separate placentas, while MZ MC twins share a single placenta [19]. It has been shown that the degree of DNA methylation dissimilarity varies between MZ MC and MZ DC twins [20], indicating that, for epigenetic purposes, one should either study MZ MC or MZ DC twins. Due to placental blood vessel connections and unequal sharing of the placenta, imbalanced blood and nutrient supply is more common in MZ MC compared to MZ DC twins [21]. Poor prenatal conditions experienced by only one co-twin often result in large intra-pair birth weight differences within MZ MC pairs [22], turning them into a 'natural experiment' to study the fetal programming origins of late onset human diseases.

We hypothesized that if poor prenatal conditions induce changes in DNA methylation patterns that remain throughout life, these changes should be visible in MZ MC twins discordant for birth weight, irrespective of their health status (degree of insulin resistance, obesity, and so on) in adulthood. To identify loci that are differentially methylated due to poor prenatal conditions, we performed an epigenome-wide association study (EWAS) in 17 adult female MZ MC twin pairs with a relative birth weight difference greater than 20%. The twins were recruited from the East Flanders Prospective Twin Survey (EFPTS), which started in 1964 and is unique due to its long-term extensive collection of perinatal (for example, birth weight, gestational age, parity) and placental data (for example, chorionicity) of nearly 8,800 twin pairs [19]. DNA was isolated from saliva, a bio-fluid that is easily accessible via a totally non-invasive method and therefore widely used in large cohort studies and perfect for diagnostic purposes. Genome-wide DNA methylation profiles were determined using the Infinium HumanMethylation450 BeadChip and validated using targeted deep coverage bisulfite sequencing. Additionally, repetitive element methylation levels were determined using methylation-dependent primer extension assays. Our thorough DNA methylation analyses in saliva of birth weight discordant MZ MC twins show that the adverse prenatal growth conditions experienced by the smaller co-twins do not lead to long-lasting DNA methylation changes in cells composing saliva (that is, buccal epithelium and leukocytes), detectable with up-to-date technologies. In addition, we observe that EWASs can be hampered by variation in cellular composition, which can lead to spurious results. We present an adjustment method to normalize the DNA methylation data with respect to variable cell-type content.

## Results

### Phenotypic characteristics of the discordant MZ MC twins

Perinatal, maternal and adult phenotypic characteristics of the 17 spontaneously conceived MZ MC female twins discordant for birth weight are presented in Table 1. Compared to the heavier co-twins, the birth weight of the lighter co-twins was, on average, 698 gram (26.7%) lower (*P *< 0.0001), with absolute and relative intra-pair birth weight differences ranging from 500 to 1,000 gram and from 21.3 to 35.7%, respectively. In addition, the frequency of a (para)central umbilical cord insertion was significantly higher in the heavier co-twins (*P *= 0.008). The mean age of the twins when the saliva samples were taken was 34.4 years, the youngest twin pair was 22 years old and the oldest 45 years. The adult phenotypic characteristics body height, body weight and body mass index did not differ between the discordant twins (*P *> 0.05). In the questionnaires, none of the twins reported that they experienced diabetes, cancer, cardiovascular or cerebrovascular disease events.

**Table 1:** Perinatal, maternal and adult phenotypic characteristics of the female MZ twins discordant for birth weight

Characteristic	Heavier co-twins	Lighter co-twins	Range	*P* ^a^
N	17	17		
				
**Perinatal**				
Gestational age (weeks)^b^	37.9 ± 2.4	37.9 ± 2.4	(34-42)	
Birth weight (g)	2,619 ± 319	1,921 ± 278	(1,440-3,100)	<0.0001
Umbilical cord insertion^b^				
(Para)central	12 (75%)	4 (25%)		
(Para)marginal	4 (25%)	9 (56%)		
Velamentous	0 (0%)	3 (19%)		0.008
				
**Maternal**				
Maternal age (years)	26.9 ± 5.4	26.9 ± 5.4	(18-43)	
Parity	1.8 ± 1.0	1.8 ± 1.0	(1-4)	
				
**Adult**				
Age (years)	34.4 ± 7.1	34.4 ± 7.1	(22-45)	
Body height (cm)	166.9 ± 6.1	165.5 ± 6.7	(155-177)	0.13
Body weight (kg)	62.7 ± 12.3	61.2 ± 14.2	(47.5-102)	0.18
Body mass index (kg/m^2^)	22.5 ± 3.8	22.3 ± 4.5	(16.7-33.7)	0.60

Continuous data expressed as mean ± standard deviation. Categorical data expressed as number of observations (%). ^a^Heavy versus light calculated using a paired *t*-test for continuous data and Fisher's exact test for categorical data. ^b^Missing for one twin pair.

### Exploratory analysis of the Infinium methylation profiles

**Figure 1:**
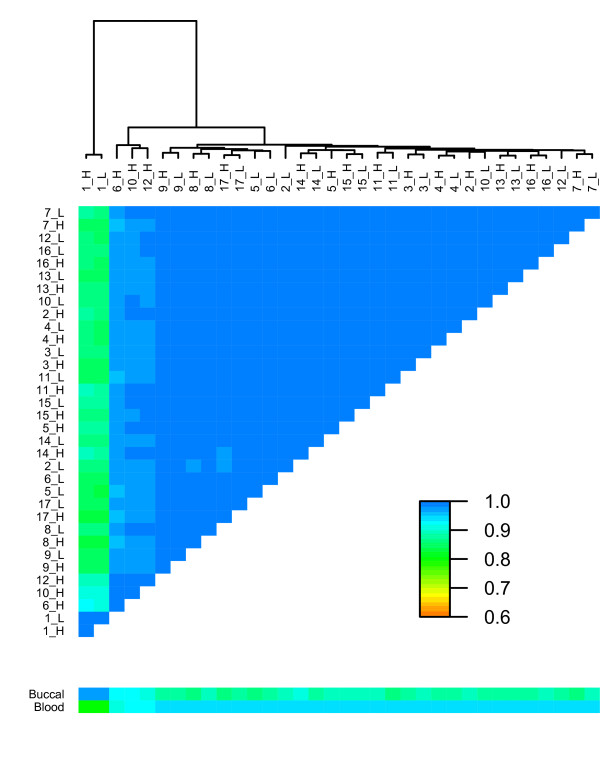
**Heatmap representing the pair-wise correlations for each pair of samples plus the reference dataset for whole-blood and buccal (27K), calculated from approximately 25,978 CpGs**. H = high birth weight, L = low birth weight.

Genome-wide DNA methylation profiles of the 17 MZ MC twins were established using the Infinium HumanMethylation450 BeadChip assay. After quality control and filtering, methylation data for 478,096 CpG sites were available. In order to identify global DNA methylation changes across the samples, the Infinium methylation data were used to calculate pair-wise array-wide Pearson correlation coefficients for each pair of samples. As depicted in Figure 1 and in Figure S1 in Additional file 1, twin pair 1 showed severe genome-wide DNA methylation changes compared to all other samples, resulting in a relatively low correlation to other samples (r = 0.846 to 0.930) while the intra-pair correlation of this twin pair was high (r = 0.996). In addition, the overall methylation profiles of samples 6_H, 10_H and 12_H deviated such that intra-pair correlation coefficients (r = 0.975-0.989) for the pairs 6, 10 and 12 were low compared to otherwise constantly high intra-pair correlation coefficients for all other twin pairs (r = 0.992 to 0.997). Careful analysis of the sample-independent and sample-dependent Infinium methylation control probes present on the BeadChip (Figures S2 and S3 in Additional file 1) revealed that the aberrant methylation profiles observed for some of the samples are unlikely the result of technical failure.

### Cellular composition of saliva as a cause of aberrant methylation profiles

Since saliva DNA is derived from leukocytes and epithelial cells [23,24], we hypothesized that the deviating DNA methylation profiles observed for some samples were at least partially attributed to inter-sample differences in cell type proportions. We therefore compared our data to genome-wide DNA methylation reference datasets obtained from whole blood and buccal epithelial cells (HumanMethylation27 BeadChip), respectively. Indeed, in contrast to all other samples, the five samples with the most deviating profiles (1_H, 1_L, 6_H, 10_H and 12_H) showed lower array-wide correlation coefficients to the reference dataset for whole blood (r_norm _= 0.943 to 0.951 versus r_deviant _= 0.812 to 0.939) than to the buccal epithelium reference dataset (r_norm _= 0.875 to 0.915 versus r_deviant _= 0.926 to 0.975) (Figure 1; Figure S4 in Additional file 1). This suggests that the deviating methylation profiles observed for these samples were a consequence of cellular composition differences - that is, a higher amount of buccal epithelial cells in the respective saliva samples.

Based on this finding we assumed that individual DNA methylation markers specific for buccal epithelium (or conversely for whole blood) can be used to determine the relative amount of buccal epithelium-derived DNA present in the samples. To illustrate that this is a valid assumption, we performed an experiment with *in vitro *generated series of two cell type mixtures profiled on the Infinium HumanMethylation450 BeadChip. The results showed that the methylation values of CpGs that are differentially methylated between the two cell types provided a good estimate of the mixed proportions (Figure S5 in Additional file 1).

Subsequently, we screened for CpGs that were highly discriminatively methylated between blood and buccal (see Supplemental methods in Additional file 1 for details) and selected the top ten most discriminatively methylated CpGs. One of them was cg18384097 in *PTPN7 *(protein tyrosine phosphatase non-receptor type 7; β-value_Buccal _= 0.82 and β-value_Blood _= 0.05), a gene preferentially expressed in hematopoietic cells (Entrez Gene ID 5778). When correlating the methylation values of every CpG with the methylation levels of the *PTPN7 *CpG, we observed that 58,987 CpGs were strongly correlated with the *PTPN7 *CpG (|r| > 0.8) and 134,265 CpGs were moderately correlated (|r| = 0.4-0.8) (Figure S6 in Additional file 1). This indicates that, for a large number of CpGs, a great amount of the observed variation in DNA methylation levels could be explained by variation in cellular composition of the saliva samples.

### Adjustment for cell type heterogeneity

Following this observation, we decided to diminish the confounding effect of the saliva cell type composition using linear regression. The detailed procedure is described in Additional file 1. In brief, first a model-fitting approach was used to select, out of the top ten most discriminatively methylated CpGs, a marker CpG of which the methylation measurements behave the most linear with respect to the changing cell proportions. In our data the *PTPN7-*associated CpG (cg18384097) gave the best linear fit to the biggest number of CpG positions, and was therefore selected as the quantitative marker measuring the proportion of buccal epithelium. Subsequently, this CpG was used to adjust the Infinium data so that the methylation level at each probe becomes linearly independent of the cellular composition in the studied sample. Noteworthy, this procedure adjusted only those CpGs that were significantly affected by the saliva composition (based on the marker model fit), while the methylation levels of the other CpGs (45%) remained unchanged. Due to the extremely high buccal epithelium content in the saliva samples of pair 1, some CpGs showed extreme values that could not be approximated by the linear model and greatly affected the regression slopes. Therefore, we excluded pair 1 from the analysis. Interestingly, pair 1 comprised the only current smokers in the sample and the only individuals with an intensive and long smoking history (>10 cigarettes/day for 25 years). This most probably caused the highly different cell composition in their saliva. The methylation data of the remaining 32 samples was adjusted for the buccal epithelium content (the pair-wise correlations for each pair of samples of the adjusted data are graphically shown in Figure S7 in Additional file 1), confirming the robustness of our approach.

### Birth weight-associated methylation variable positions

**Figure 2:**
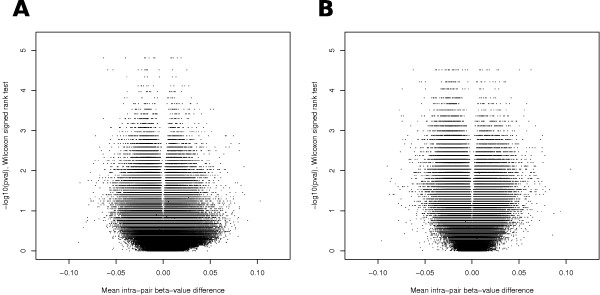
**Volcano plots of the distributions of the *P*-values resulting from the Wilcoxon signed-rank test versus the corresponding mean β-value difference for each CpG position**. **(a) **Unadjusted data (72 CpG sites had a *P*-value <0.01 and mean β-value difference >0.05). **(b) **Data adjusted for buccal epithelium content using the *PTPN7 *CpG (131 CpG sites had a *P*-value <0.01 and mean β-value difference >0.05; pair 1 was excluded).

Next we tested the hypothesis that poor prenatal conditions led to significant DNA methylation differences between the heavy and light co-twins for each CpG site independently using the non-parametric Wilcoxon signed-rank test. Figure 2 gives the volcano plots - that is, distributions of the resulting *P*-values versus the corresponding mean β-value difference for each CpG position, for both the unadjusted data (Figure 2a) and the data adjusted for buccal epithelium content (Figure 2b). The plot documents that large significant DNA methylation differences could not be detected between the heavy and light co-twins (upper corners of both plots are void of data points). In addition, one can also notice that the uneven cellular composition resulted in a considerable amount of CpGs that had relatively big effect sizes but high (non-significant) *P*-values. This confirms that the vast majority of the changes were not associated with birth weight, but were the result of within-pair variation in cellular composition. We therefore proceeded with the results of the data where the affected CpG positions were adjusted for buccal epithelium content variation.

Given the small number of significant changes, we selected a non-stringent significance threshold (uncorrected *P*-value <0.01). In addition, to identify CpG sites likely to be validatable using other methods [25], we focused on CpG positions that showed an absolute mean β-value difference >0.05. Out of 478,096 sites, 7,859 CpGs had a *P*-value <0.01 with absolute mean β-value differences ranging from 0.0012 to 0.1049 and *P*-values ranging from 0.0092 to 3.05 × 10^-5^. Only 131 of these CpGs showed an absolute mean β-value difference >0.05. To exclude the potential influence of other blood-derived cells present in saliva [26], we extended the adjustment model to include markers of leukocyte subtypes (that is, neutrophils, B-lymphocytes, CD4+ T-lymphocytes, CD8+ T-lymphocytes and natural killers) (see Supplemental methods and Tables S1 and S2 in Additional file 1 for details). In total, 3,153 CpGs remained significant (*P *< 0.01) in both analyses, of which only 45 CpGs showed an absolute mean β-value difference >0.05 (ranging from 0.05 to 0.08). We treated this set of 45 CpGs, further denoted as 'birth weight-associated methylation variable positions' (BW-MVPs), as being most likely differentially methylated between the discordant MZ twins, regardless of the cellular composition (Table S3 in Additional file 1). Subsequently, we tested these BW-MVPs using state-of-the-art technical validation to prove or disprove that they are true biological effects.

### BW-MVP validation using deep bisulfite sequencing

The 45 BW-MVPs were prioritized for independent DNA methylation validation either based on their biological significance, regulatory relevance and/or whether neighboring probes were also differentially methylated. In total, eight BW-MVPs were validated using deep bisulfite sequencing (DBS) and their functional characteristics are summarized in Table 2. Some of the prioritized BW-MVPs were situated in genes strongly involved in glucose and/or lipid metabolism or have been implicated in T2D or obesity risk (for example, *APPL2*, *IGF2BP2*, *PHKG2 *and *PPARGC1B*), which is in line with the observation that low birth weight is associated with increased metabolic disease risk. Of the eight selected loci, only three were affected (*IGF2BP2*, *PAPOLA *and *PPARGC1B*) by buccal content, so the methylation values of the other five remained unchanged. Moreover, in the unadjusted data the *IGF2BP2 *and *PPARGC1B *CpGs were also significantly differentially methylated between the discordant twins. To be able to adjust the DBS data of the *IGF2BP2*, *PAPOLA *and *PPARGC1B *CpGs for buccal epithelium content, the *PTPN7 *CpG (buccal epithelium marker) was also analyzed using DBS and simultaneously served as a positive control. High quality DBS data were obtained for all amplicons, with an average sequencing coverage of 988 reads (that is, individual chromosomal patterns) per amplicon per sample. Examples of the methylation profiles obtained by DBS are given in Figure S8 in Additional file 1.

**Table 2:** Characteristics of the eight BW-MVPs that were prioritized for further validation using DBS

CpG number	Region	Gene/ element	Full name	Function
cg14123607	Intron 1/insulator	*APBA1*	Amyloid beta A4 precursor protein-binding family A member 1	Associated with reduced production of amyloid-β peptide, which is considered a key player in Alzheimer's disease [52]
cg12170649	Intron 2/enhancer	*APPL2*	Adaptor protein, phosphotyrosine interaction, PH domain and leucine zipper containing 2	Involved in cell proliferation and embryonic development. Acts as negative controller of adiponectin signaling and SNPs in *APPL2 *have been associated with obesity [53-55]
cg26404226	Enhancer	Chr10q23.3		
cg15487251^a^	Promoter	*IGF2BP2*	Insulin-like growth factor 2 mRNA-binding protein 2	mRNA binding protein involved in RNA localization, stability and translation. Intronic SNP in *IGF2BP2 *has been identified as T2D risk factor by several GWAS [56]
cg10362113^a^	Intron 1/weak enhancer	*PAPOLA*	Poly(A) polymerase alpha	Plays a predominant role in addition of the 3'-poly(A) tail to mRNA precursors, which is important for mRNA stability, transport and translation [57]
cg02409150	Intron 4	*PHKG2*	Phosphorylase kinase, gamma 2 (testis)	Encodes the gamma subunit of the liver-specific phosphorylase kinase, which activates the enzyme glycogen phosphorylase, resulting in glycogen breakdown [58]
cg15049370^a^	Intron 2/enhancer	*PPARGC1B*	Peroxisome proliferator-activated receptor gamma coactivator 1-beta	Multifunctional transcriptional co-regulator involved in many metabolic processes, including mitochondrial oxidative metabolism and hepatic lipogenesis [59]
cg22768222	Intron 1/weak enhancer	*RUNX2*	Runt-related transcription factor 2	Transcription factor essential for osteoblast and chondrocyte differentiation [60]

^a^Affected by buccal content. BW-MVP, birth weight-associated methylation variable position; DBS, deep bisulfite sequencing; GWAS, genome-wide association studies; SNP, single nucleotide polymorphism; T2D, type 2 diabetes.

Technical performance of both methods was first analyzed by comparing the (unadjusted) DBS data of the validated CpGs with the (unadjusted) Infinium data for every analyzed sample separately. The individual correlation plots are presented in Figure S9 in Additional file 1, and the correlation coefficients calculated for each sample are summarized in a box plot in Figure S10 in Additional file 1. Overall, the Infinium data correlates very well with the DBS data, with a median correlation coefficient of 0.97. From both figures it becomes also clear that sample 12_H is an outlier with a correlation coefficient of only 0.70. When we correlate the (unadjusted) Infinium data with the (unadjusted) DBS data for each validated CpG site separately (Table 3; Figures S11 and S12 in Additional data file 1), the DBS data of the CpG sites in *APBA1*, *APPL2*, *PHKG2*, *PPARGC1B *and *RUNX2 *correlate poorly with the Infinium data (r ≤ 0.60). For the other four CpG sites (Chr10q23.3, *IGF2BP2*, *PAPOLA *and *PTPN7*) the correlation coefficients were higher (r = 0.73-0.91) and, as expected, the highest correlation was observed for the buccal epithelium marker (*PTPN7*).

**Table 3:** Validation of eight BW-MVPs and the *PTPN7 *CpG using DBS in the 17 discordant MZ twins

		Infinium			DBS				
						
CpG number	Gene	Mean^a ^± SD	Range		Mean^b ^± SD	Range	Mean number of reads (range)	r_Pearson_	*P*
cg14123607	*APBA1*	0.19 ± 0.08	0.06-0.43		0.09 ± 0.05	0.04-0.24	950 (440-1670)	0.61	0.0001
cg12170649	*APPL2*	0.83 ± 0.05	0.71-0.91		0.97 ± 0.02	0.93-0.99	1,120 (785-1,725)	-0.23	0.18
cg26404226	NA	0.52 ± 0.07	0.38-0.72		0.35 ± 0.08	0.21-0.54	1,201 (716-2,345)	0.73	<0.0001
cg15487251	*IGF2BP2*	0.57 ± 0.08	0.30-0.69		0.55 ± 0.10	0.24-0.68	1,156 (531-1,824)	0.80	<0.0001
cg10362113	*PAPOLA*	0.83 ± 0.12	0.41-0.98		0.84 ± 0.16	0.25-0.95	997 (188-1,689)	0.87	<0.0001
cg02409150	*PHKG2*	0.91 ± 0.05	0.80-0.98		0.93 ± 0.02	0.87-0.97	749 (294-1,301)	0.37	0.03
cg15049370	*PPARGC1B*	0.78 ± 0.07	0.62-0.98		0.96 ± 0.02	0.93-1.00	1,019 (522-1,792)	0.32	0.06
cg18384097^c^	*PTPN7*^c^	0.40 ± 0.17	0.16-0.89		0.28 ± 0.20	0.09-0.98	848 (383-1,334)	0.91	<0.0001
cg22768222	*RUNX2*	0.32 ± 0.09	0.05-0.55		0.20 ± 0.05	0.02-0.26	853 (302-1,347)	0.55	0.0008

Pearson's correlation coefficients (r_Pearson_) between the (unadjusted) Infinium and the (unadjusted) DBS data are given as well as the overall mean methylation, standard deviation (SD) and the overall methylation range observed for the Infinium and the DBS data. ^a^Infinium data (unadjusted) are expressed as mean β-value ± SD. ^b^DBS data (unadjusted) are expressed as mean methylation level ± SD, where the methylation level is calculated by dividing the number of reads in which the particular CpG is methylated by the total number of sequenced reads. ^c^Buccal marker. 'Mean number of reads (range)' is the average number of sequenced bisulfite reads per amplicon (minimum to maximum number of sequenced bisulfite reads per amplicon). *P *= *P*-value under H_0_: r_Pearson _= 0. BW-MVP, birth weight-associated methylation variable position; DBS, deep bisulfite sequencing; MZ, monozygotic.

Subsequently, we tested whether the significant DNA methylation differences between the heavy and light co-twins at these BW-MVPs obtained using the Infinium data could be confirmed with the DBS data. We again performed a Wilcoxon signed-rank test and the results of both technologies are presented in Table 4. Unfortunately, in the DBS data no significant DNA methylation differences were observed between the heavy and light co-twins (*P *> 0.01). Moreover, in the DBS data we can also include CpGs neighboring the selected MVPs and also for those CpGs no significant methylation differences between the heavy and light co-twins were observed (data not shown). Since the correlation analysis revealed that sample 12_H suffers from technical problems (Figure S9 in Additional file 1), we repeated the analysis without pair 12. Still, in the DBS data no significant differences between the heavy and light co-twins were observed, while the differences in the Infinium data remained significant.

**Table 4:** Differential methylation analysis of the eight selected BW-MVPs using the Infinium and DBS data

		Infinium					DBS			
				
CpG number	Gene	Mean heavy co-twins^a^	Mean light co-twins^a^	Mean difference	*P* ^b^		Mean heavy co-twins^c^	Mean light co-twins^c^	Mean difference	*P* ^b^
cg14123607	*APBA1*	0.22 ± 0.07	0.15 ± 0.07	0.07 ± 0.05	0.0008		0.09 ± 0.05	0.09 ± 0.05	0.006 ± 0.04	0.30
cg12170649	*APPL2*	0.81 ± 0.04	0.86 ± 0.02	-0.06 ± 0.05	<0.0001		0.97 ± 0.02	0.97 ± 0.02	-0.002 ± 0.02	0.86
cg26404226	NA	0.50 ± 0.05	0.56 ± 0.07	-0.05 ± 0.04	<0.0001		0.35 ± 0.08	0.36 ± 0.06	-0.01 ± 0.05	0.46
cg15487251	*IGF2BP2*^d^	0.53 ± 0.05	0.58 ± 0.04	-0.05 ± 0.05	0.002^e^		0.65 ± 0.05	0.64 ± 0.06	0.01 ± 0.03	0.04
cg10362113	*PAPOLA*^d^	0.83 ± 0.08	0.77 ± 0.06	0.06 ± 0.07	0.008		0.98 ± 0.04	0.99 ± 0.03	-0.01 ± 0.05	0.50
cg02409150	*PHKG2*	0.88 ± 0.05	0.94 ± 0.03	-0.06 ± 0.05	<0.0001		0.93 ± 0.02	0.93 ± 0.02	-0.006 ± 0.03	0.43
cg15049370	*PPARGC1B*^d^	0.70 ± 0.06	0.77 ± 0.07	-0.07 ± 0.07	0.002^e^		0.96 ± 0.01	0.97 ± 0.02	-0.007 ± 0.02	0.23
cg22768222	*RUNX2*	0.37 ± 0.07	0.31 ± 0.05	0.06 ± 0.07	0.008		0.21 ± 0.03	0.21 ± 0.03	0.001 ± 0.05	1.00

Results of the Wilcoxon signed-rank test of the 8 selected MVPs performed on the Infinium and the DBS data in the 16 MZ twins discordant for birth weight (pair 1 was excluded) are presented. ^a^Infinium data are expressed as mean β-value ± SD. ^b^Heavy versus light calculated using a Wilcoxon signed-rank test. ^c^DBS data are expressed as mean methylation level ± SD, where the methylation level is calculated by dividing the number of reads in which the particular CpG is methylated by the total number of sequenced reads. ^d^Infinium and DBS data were adjusted for buccal content (using *PTPN7*) and the adjusted values are presented. ^e^Also significant without adjusting for buccal marker. Mean difference = Heavy co-twin - Light co-twin. BW-MVP, birth weight-associated methylation variable position; DBS, deep bisulfite sequencing; MZ, monozygotic.

### HNF4A methylation

In addition, we analyzed the hepatocyte nuclear factor 4 alpha (*HNF4A*) promoter, which has previously been identified as differentially methylated between IUGR neonates and controls in a genome-wide scan [16]. Since the significant region was not covered by the Infinium chip, we analyzed it using DBS with a mean sequence coverage of 865 reads ranging from 176 to 1,324 reads per sample. The *HNF4A *methylation levels correlated significantly with the buccal epithelium marker *PTPN7*, and therefore the *HNF4A *methylation data were adjusted for *PTPN7 *methylation as described earlier. Following such an adjustment, none of the 10 CpG sites within the *HNF4A *amplicon retained significant methylation differences between the discordant twins (*P *> 0.01, data not shown).

## Global DNA methylation analysis on repetitive elements

Finally, the DNA methylation levels of the genome dispersed repetitive elements human endogenous retrovirus type K (*HERVK*) and long interspersed nuclear element-1 (LINE1) were evaluated using methylation-dependent primer extension assays. For every CpG analyzed, mean methylation indices (MIs; similar to Illumina's β-values) were very similar among the heavy and light co-twins and no significant differences were observed (*P *> 0.05) (Table 5). Some CpGs strongly correlated with *PTPN7 *methylation (*HERVK *CpG1 r = -0.89, LINE1 CpG1 r = -0.49), indicating that global methylation levels are lower in buccal epithelium. Accordingly, when we repeated the analysis following *PTPN7 *methylation adjustment, no significant associations could be detected (data not shown).

**Table 5:** Methylation analysis of *HERVK *and LINE1 in the 16 discordant MZ twins (pair 1 excluded)

Element	CpG^a^	Mean MI heavy co-twins	Mean MI light co-twins	Mean MI difference	*P* ^b^
*HERVK*	1	0.61 ± 0.05	0.63 ± 0.02	-0.02 ± 0.06	0.38
	2	0.36 ± 0.01	0.36 ± 0.01	0.0008 ± 0.006	0.86
LINE1	1	0.58 ± 0.02	0.58 ± 0.02	-0.0007 ± 0.01	0.86
	2	0.37 ± 0.02	0.38 ± 0.02	-0.004 ± 0.02	0.50

All procedures (including bisulfite treatment, PCR and single-nucleotide primer extension assays in combination with ion-pair reversed-phase high-performance liquid chromatography separation technique (SIRPH) assays) were performed in duplicate; the mean values were used for the statistical analysis. Data are expressed as mean MI ± standard deviation. MI, methylation index; MI difference, MI heavy co-twin - MI light co-twin. ^a^CpG 1 and 2 corresponds to the CpG tagged by SNuPE primer 1 and 2, respectively. ^b^Heavy versus light calculated using a Wilcoxon signed-rank test.

## Discussion

We aimed to identify loci that remain differentially methylated in adult body fluid cells as a consequence of a poor prenatal environment. Our hypothesis was that DNA methylation changes induced by adverse intra-uterine conditions are detectable in adult MZ MC twins with large intra-pair weight differences at birth, irrespective of their health status in adulthood. We used Infinium HumanMethylation450 BeadChip to profile DNA methylation changes genome-wide, and applied 454 GSFLX-based single-molecule DBS to validate potential methylation variable positions (MVPs). To assess possible changes in repetitive element methylation, we applied bisulfite-based primer extension high-performance liquid chromatography (SIRPH) assays. Despite cellular composition differences, our thorough DNA methylation analyses show that the methylomes in saliva of birth weight discordant MZ MC twins are very similar.

All analyses were performed on DNA isolated from saliva, a bio-fluid that contains adequate amounts of DNA and is easy accessible via a totally non-invasive method. Assuming that MVPs are maintained in a systemic way, saliva DNA should be suitable for diagnostic and prognostic purposes, like any other accessible body fluid such as blood. However, we observed that the composition of saliva can be highly variable, possibly causing confounding effects since DNA methylation signatures are cell type-specific. Such hardly controllable effects should be accounted for when studying the association of DNA methylation to the phenotype of interest. This can either be done by separating cells prior to the methylation analysis, which is usually a challenging experimental task, or by using methods that allow a post-sampling adjustment for cellular composition. Indeed, here we show that cell type-specific epigenetic signatures of cells can be used for such post-sampling adjustment.

When (young) MZ twins are used for epigenetic studies, tissues other than blood are preferred (often buccal epithelium). This is because MZ twins often have a shared blood supply during intra-uterine development, and therefore epigenetically discordant MZ twins can display the same epigenetic defect in blood while, for instance, in fibroblasts the epigenetic defect is restricted to the affected twin only [27,28]. Interestingly, Kaminsky *et al. *[20] observed that methylation profiles of buccal swab DNA are significantly more variable within MZ MC twins compared to MZ DC twins. They suggested that this epigenetic dissimilarity may reflect differences in epigenetic divergence among embryonic cells at the time of splitting. Since buccal swabs also contain saliva [24], our results indicate that the previously reported epigenetic differences within MZ MC twins observed by Kaminsky *et al. *[20] might be caused by sample composition-attributed variation of, for example, leukocytes and epithelial cells [24], rather than a real developmental difference.

Our results show that MZ twins have very similar genome-wide DNA methylation profiles. After controlling for sample composition-attributable variation, we obtained 3,153 CpGs that were differentially methylated between the heavy and light co-twins with nominal significance (*P *< 0.01), of which only 45 CpGs showed an absolute mean β-value difference >0.05. To verify whether these loci were true BW-MVPs, 8 of these 45 loci were validated using state-of-the-art targeted DBS. When correlating the Infinium with the DBS data for each individual separately, the two technologies gave consistent results and the correlations were high. However, the DBS data did not replicate the DNA methylation differences between the heavy and light co-twins. Nevertheless, when correlating the Infinium with the DBS data for each validated CpG site separately, we observed a wide range of Pearson correlation coefficient values. The highest correlation was observed for the CpG in the buccal epithelium marker *PTPN7 *(r = 0.91), which served as a positive control, and the buccal content-affected *PAPOLA *and *IGF2BP2 *CpGs (r = 0.87 and r = 0.80, respectively) (Table 3). This indicates that true biological variation, linked to the variation of the cell type proportions in saliva samples, is confirmed by DBS. On the other hand, the decreasingly significant correlation values observed for the remaining CpGs indicates low or absent true biologically meaningful variation in the measured DNA methylation levels. The fact that DBS did not replicate the differences between heavy and light co-twins might thus indicate that the few BW-MVPs identified using the HumanMethylation450 assay are the result of technical noise, that is, false positives. This is coherent with the fact that if a more stringent significance criteria had been used to correct for multiple testing, none of the BW-MVPs would have been called significant. In addition, the DAVID tool did not identify enrichments in any of the numerous functional annotation categories for the genes underlying the BW-MVPs [29], indicating that there was no evidence of coordinated DNA methylation changes at BW-MVPs that would reflect potential regulation events in groups of loci.

Compared to other widely used genome-wide methylation profiling technologies that are based on methylation-sensitive restriction digestion (HELP (HpaII tiny fragment Enrichment by Ligation-mediated PCR), CHARM) or affinity-based enrichment (MeDIP, MethylCap), the Infinium assay has a higher resolution (single base pair) and therefore expected to have a higher sensitivity [30]. However, whether the Infinium assay is sensitive enough to distinguish between an absolute mean β-value difference of approximately 0.05 to 0.07 is unclear. Bibikova *et al. *[31] estimated that with the Infinium HumanMethylation27 BeadChip, on average, β-value differences of 0.14 or larger can be detected, with a higher sensitivity at unmethylated and fully methylated sites (for example, at unmethylated promoters on average β-value changes of approximately 0.07 were detectable). We made an attempt to estimate the technical noise level by examining the 64 SNP probes that are present on the chip. For the twin samples that were heterozygous, the SNP probes showed an absolute intra-pair mean β-value difference of 0.00 to 0.03. Hence, trying to replicate absolute mean β-value differences of 0.05 to 0.07 can be a realistic goal, assuming that all probes on the chip perform as well as these SNP probes. Nonetheless, this is a strong assumption and a number of technical issues are likely to undermine the performance of the HumanMethylation450 BeadChip - for example, differences between the Infinium I and II technologies, multiple CpGs in the probe sequences, cross-hybridization of (repetitive) sequences (for example, *PPARGC1B *contains *Alu *element) [32,33]. On the other hand, whether DBS, which is currently considered as the gold standard, is sensitive enough to replicate a 5% methylation difference is also questionable. On average, we obtained 988 high quality reads per sample per amplicon; thus, the lack of replication is unlikely to be the result of low quality DBS data. Still, in three of the sequenced amplicons informative SNPs were present and for the heterozygous twins a mean absolute intra-pair allele frequency difference of 0.05 to 0.08 was observed. This indicates that DBS also suffers from technical variation, which is probably the result of random bias induced by PCR amplification. Since all currently used diagnostic methods are PCR-based, focusing on small methylation differences might currently not be worthwhile. These aspects should be more carefully considered in EWASs. Taken together, we cannot exclude the possibility that the BW-MVPs identified in this study are false positives. The fact that the detected differences are on the border of technical variation makes it unlikely that they can be regarded as biologically significant.

The first EWAS for birth weight was performed on CD34+ hematopoietic stem cells from cord blood of five IUGR neonates and five controls using the HELP assay and identified moderate changes at 56 loci [16]. The authors validated only one locus, the *HNF4A *promoter, using another technology (bisulfite MassArray). We also analyzed this locus using DBS, but observed no significant differences between the discordant twins. Since the authors studied CD34+ hematopoietic stem cells from cord blood, our negative results might indicate that the changes they observed do not maintain until adulthood or that they are specific for CD34+ hematopoietic stem cells. On the other hand, they only observed a 6% methylation difference between IUGR neonates and controls [16], which remains according to our technical observations difficult to replicate.

Currently, six genome-wide DNA methylation studies for birth weight have been published, the details of which are presented in Table 6[16,34-38]. All of them studied fetal tissues - umbilical cord blood, umbilical vascular endothelial cells and/or placenta. One study used the HELP assay [16], while the other five studies used the HumanMethylation27 BeadChip. In addition, one of the studies [38] also used a twin design (18 MZ and 10 DZ), although the authors did not select the twins based on birth weight discordancy and therefore the mean relative intra-pair birth weight difference of the MZ twins included in their study was only 10.1%. All these EWASs for birth weight reported a number of differentially methylated loci. However, only one gene (*PRSS3*) was reported by more than one study [16,34] and none of the loci identified in these studies was significant in our analysis. Moreover, none of these studies performed an intense technical validation comparable to the one made in our study or controlled for sample composition-attributed variation. In summary, while the number of potential candidate loci that become differentially methylated due to an adverse prenatal environment is rapidly growing, the validity of these effects, perhaps with the exception of *PRSS3 *that was reported by two studies, is questionable since there is no overlap between the reported loci.

**Table 6:** Genome-wide DNA methylation studies for birth weight

Study	Design	Sample	Tissue	Method	Significant loci	Remark
Einstein *et al. *[16]	Population based	5 IUGR and 5 AGA	CD34+ hematopoietic stem cells (cord blood)	HELP	56 loci (*P *< 0.00001)	
Banister *et al. *[37]	Population based	89 IUGR and 117 AGA	Placenta	HM27	22 loci (number predetermined)	
Fryer *et al. *[34]	Population based	12 newborns	Cord blood	HM27	304 loci (*P *< 0.05)	Samples were selected to give a range in LINE1 methylation values
Adkins *et al. *[36]	Population based	201 newborns	Cord blood	HM27	10 loci (*P *< 0.001)	
Turan *et al. *[35]	Population based	48 newborns	Cord blood and placenta	HM27	23 loci	Regularized regression model fit was used (R^2 ^> 0.80)
Gordon *et al. *[38]	Twin design	18 MZ and 10 DZ^a^	CBMCs, placenta and UVECs	HM27	7 loci in DZ CBMCs 1 loci in MZ UVECs (FDR <0.1)	Twins not selected for birth weight discordancy

^a^Maximal 18 MZ and 10 DZ twins per tissue. AGA, appropriate for gestational age; CBMC, cord blood mononuclear cell; DZ, dizygotic twin; FDR, false discovery rate; HELP, HpaII tiny fragment Enrichment by Ligation-mediated PCR assay; HM27, HumanMethylation27 BeadChip; IUGR, intra-uterine growth retarded; MZ, monozygotic twins; UVEC, umbilical vascular endothelial cell.

Some studies also examined the relation between birth weight and global DNA methylation levels by assessing repetitive elements. Fryer *et al. *[34] observed in 12 cord blood samples that LINE1 methylation was higher among the heavier newborns. However, Michels *et al. *[39] observed in cord blood of 319 newborns a significant correlation between low birth weight, high birth weight and preterm birth with reduced LINE1 methylation, while in placental tissue they observed that low birth weight individuals had higher LINE1 methylation compared to normal birth weight individuals. In addition, Wilhelm-Benartzi *et al. *[40] observed in 184 placenta samples a positive association between LINE1 and AluYb8 methylation and birth weight. We did not observe any significant differences in LINE1 and *HERVK *methylation between the heavy and light co-twins, but we observed differences in LINE1 and *HERVK *methylation between leukocytes and epithelial cells. Further studies should consider such sample composition-attributed variation as it might be responsible for the inconsistent reports concerning global DNA methylation and intra-uterine growth.

The majority of the genome-wide methylation studies for birth weight published thus far used a population-based design [16,34-37]; thus, their outcome variable birth weight suffers from variation induced by gestational age, gender, maternal factors (for example, maternal weight, age, parity) and, most importantly, genetic differences. For all these factors our twin study is controlled and hence methylation variations associated with them should be eliminated. Since we fail to identify any major methylation change, the contribution of such variable factors in data interpretation should be considered more carefully. One might argue that our negative outcome is the result of the shared intra-uterine blood supply of MZ MC twins, which 'diluted' any differential methylation signals. Nevertheless we are aware of this problem and focused on adult twins, since we earlier observed that epigenetic discordance in MZ MC twins, even those that suffered from twin-to-twin transfusion syndrome, becomes measurable in saliva when they grow older [27]. Moreover, for twin pair 2 it was recorded that they suffered from twin-to-twin transfusion syndrome *in utero*. If the severely unbalanced intra-uterine blood flow did have an impact on their leukocyte populations, then for this pair one would expect to see a higher intra-pair correlation, which was not higher than expected on average (Figure S7 in Additional file 1).

Since none of the twins reported in the questionnaire suffer from acute diabetes, cancer, cardiovascular or cerebrovascular diseases, one might reason that our approach enriched for healthy individuals. However, the twins were only recruited based on being very discordant for birth weight and adult health status was never used as an inclusion criterion. In addition, the EFPTS is a prospective and population-based twin registry [19]. Therefore, neither our selection strategy nor the EFPTS ever enriched for healthy individuals. In addition, a medical examination was not conducted for this study, so the actual adult health status of the twins is unknown. Our twin sample is also relatively young and clear symptoms are expected to appear at later ages. Moreover, manifestation of metabolic disorders is strongly related to lifestyle factors.

Finally, through the *post hoc *power calculation presented in Table S7 in Additional file 1, we demonstrate that our approach has sufficient power to detect an absolute mean β-value difference of at least 0.05. To exclude that our negative study outcome is the result of a high false negative rate, we applied a nominal significance threshold of 0.01 (gives approximately 99% power). Note that the statistical analyses show that our design can also easily detect smaller methylation differences, since 3,108 of the 3,153 CpGs having a *P *< 0.01 in the final analysis showed an absolute mean β-value difference below 0.05. However, these small differences are not reproducible using DBS and thus remain in the range of technical inaccuracy.

## Conclusions

Our study is based on the assumption that methylation changes caused by a poor prenatal environment remain throughout life in many cell types (systemic). The fact that we used saliva instead of whole blood is, in this respect, an advantage since saliva contains ectoderm- and mesoderm-derived cells, while blood contains only the latter. Nevertheless, our negative results might indicate that the methylation differences are restricted to biologically relevant metabolic tissues (for example, pancreas, liver, muscle, adipose tissue) and thus absent in cells composing saliva. It is also possible that the methylation differences are temporary and are not maintained into adulthood. Due to placental blood vessel connections, blood of young MZ MC birth weight discordant twins is not suitable for epigenetic studies. Studying young MZ DC twins would be an alternative as they do not experience intra-uterine vascular connections, but they are more rare (33% of all MZ twins) and have smaller intra-pair birth weight differences. On the other hand, the HumanMethylation450 BeadChip covers just approximately 2% of all CpGs in the genome and gene bodies and regulatory intergenic regions are underrepresented on the chip. In addition, birth weight discordancy in MZ MC twins can arise from different pathologies and in this respect our group is certainly not homogeneous (for example, different locations of umbilical cord insertion). Despite these limitations, we can conclude that genome-wide and locus specific DNA methylation perturbations are small and not abundant in cells composing saliva (that is, epithelium and leukocytes) of individuals that experienced severe intra-uterine growth restriction.

## Material and methods

### Participants

For this study, 17 spontaneously conceived MZ MC female twin pairs discordant for birth weight were recruited from the EFPTS [19], which is a population-based twin register that started in 1964 and recorded all multiple births in the Belgian Province of East Flanders until the present. Discordancy is defined as relative birth weight difference ≥20% ([Highest birth weight - Lowest birth weight]/Highest birth weight), with the lightest twin having a birth weight below the 10th percentile and the heavier twin having a birth weight between the 10th and 90th percentiles for that gestational age, gender, parity and chorion type (based on twin-specific growth charts [41]). To minimize variation due to gender-specific methylation differences [42], only female twins were included. In addition, to assure that the DNA methylation changes remain throughout life, only adult (aged ≥18 years) discordant MZ MC female twins were included (in total, 61 pairs satisfy these selection criteria). Of the 17 twin pairs, 15 pairs were newly recruited for this study, while two pairs were previously recruited for another study [43]. None of the participants suffered from severe postnatal complications. The Ethics Committee of the Faculty of Medicine of the Katholieke Universiteit Leuven approved the project and all participants gave written informed consent. The study was conducted according to the principles of the Declaration of Helsinki.

### Phenotypes

Information on birth weight and parity was obtained from obstetric records within 24 hours after delivery. Gestational age was reported by the obstetrician and was calculated as the number of completed weeks of pregnancy based on the last menstrual period. The obstetricians and the pediatricians answered a structured questionnaire that provided, among other items, information on the mode of conception, abnormalities of the children and the health status of the children for the period they stayed in the neonatal unit. A trained midwife examined the placentas within 24 hours of delivery and assessed chorionicity macroscopically following a standardized protocol [44]. Adult phenotypic data were retrieved from a mailed questionnaire, which included self-reported items on current body weight, body height, physical activity level, medical history, smoking behavior and alcohol consumption. Body mass index was calculated as self-reported body weight divided by the square of height (kg/m^2^).

### Genomic DNA extraction

Saliva samples were collected using the Oragene DNA Self-collection Kit (DNA Genotek, Ottawa, Canada). Genomic DNA was extracted from saliva using the GenElute Mammalian Genomic DNA Miniprep Kit (Sigma-Aldrich, St Louis, MO, USA) according to the manufacturer's instructions. DNA was quantified using the Qubit fluorometer (Invitrogen GmbH, Karlsruhe, Germany) and qualified using the Nanodrop 2000C spectrophotometer (Thermo Scientific, Wilmington, DE, USA). Per DNA extraction batch, only one member per twin pair was processed.

### Zygosity confirmation

Although all twins were monochorionic and thus assumed to be monozygotic, zygosity was confirmed by genotyping 17 highly polymorphic microsatellite markers using the PowerPlex ESI 17 system (Promega Corporation, Madison, WI, USA), with an average certainty exceeding 99.99%.

### Genome-wide DNA methylation analysis

To avoid differences in methylation levels within twins due to bisulfite treatment or PCR bias, both members of a twin pair were always processed in the same batch.

#### Bisulfite treatment

For the genome-wide study, per sample 1 μg (2 × 500 ng) of genomic DNA (OD260/280 >1.8) extracted from saliva was treated with bisulfite using the EZ DNA Methylation-Gold Kit (D5005, Zymo Research, Orange, CA, USA). In brief, 700 μl water, 300 μl M-Dilution Buffer and 50 μl M-Dissolving Buffer was added to the CT Conversion Reagent tube. After mixing, 110 μl of the CT Conversion Reagent was added to 40 μl of DNA, which was then incubated for 10 minutes at 98°C followed by 3 hours at 64°C. Afterwards, bisulfite-converted DNA samples were purified by loading, desulfonating and washing on the provided Zymo-Spin™ IC columns (following the manufacturer's instructions), eluted in 12 μl M-Elution Buffer and stored at -20°C prior to processing.

#### Infinium HumanMethylation450 BeadChip

Genome-wide DNA methylation profiles were generated using Illumina's Infinium HumanMethylation450 Beadchip assay (Illumina, San Diego, CA, USA) at the Department of Psychiatry and Psychotherapy of the Saarland University Hospital. The assay allows determination of DNA methylation levels at >450,000 CpG sites covering all designable RefSeq genes, including promoter, 5', and 3' regions; it captures CpG islands and shores, non-CpG methylated sites, microRNA promoter regions and disease-associated regions identified through genome-wide association studies [45]. The Infinium Methylation Assay was performed according to the manufacturer's instructions. In brief, 4 μl of denatured bisulfite-treated DNA was isothermally amplified overnight at 37°C, followed by an enzymatic fragmentation step using end-point fragmentation. The fragmented DNA was then precipitated, resuspended and loaded on the 12-sample BeadChip (see Table S4 in Additional file 1 for the distribution of the samples across the beadchips). The chips were incubated overnight at 48°C, allowing the fragmented DNA to hybridize to the locus-specific 50-mers on the chip. Unhybridized and non-specifically hybridized DNA was washed away, followed by a single-base extension reaction using DNP- and Biotin-labeled ddNTPs. Subsequently, the hybridized DNA was washed away and a multi-layer staining process was carried out to attach fluorescent dyes to the labeled extended primers. The fluorescently stained chips were imaged using an Illumina HiScanSQ scanner and the Illumina's GenomeStudio software (Methylation Module v1.8) was used to extract the data, subtract the background and to normalize the data using internal controls present on the chip (see Supplemental methods in Additional file 1 for details). The overall performance of the normalization procedure is illustrated in Figure S13 in Additional file 1. Subsequently, for each CpG site a β-value was calculated, which represents the fraction of methylated cytosines at that particular CpG site (0 = unmethylated, 1 = fully methylated). Only CpGs with a detection *P*-value <0.001 in all samples were included (Table S4 in Additional file 1). In total, 4,325 out of 482,421 CpGs were excluded, of which 351 were located on the Y-chromosome.

### Deep bisulfite sequencing analysis

Selected MVPs were validated using DBS. As long as the stocks lasted, the bisulfite DNA used for the genome-wide scan was used for validation analysis or a new bisulfite treatment was performed using standard protocols. In brief, 2 M sodium bisulfite and 0.6 M NaOH was added to 300 ng genomic DNA, which was then incubated for 15 minutes at 99°C and 30 minutes at 50°C, followed by 2 cycles of 5 minutes at 99°C and 90 minutes at 50°C. Afterwards, bisulfite-treated DNA was sequentially desulfonated (with 0.3 M NaOH), washed with 1× TE and recovered in 50 µl 0.5× TE using centrifugal filter units YM-30 (Millipore, Schwalbach, Germany).

Amplicons were generated using region-specific primers having on their 5' ends the recommended GS-FLX A and B adaptors sequences (Lib-L) and multiplex identifiers (MID) (Roche, Mannheim, Germany). Bisulfite PCRs were carried out in 30 µl mixes, including 1 to 3 µl bisulfite-treated DNA, 0.2 mM of each dNTP, 3 U HOT FIREPol DNA polymerase (Solis BioDyne, Tartu, Estonia), 1× reaction buffer B (Solis BioDyne), 2.5 mM MgCl_2_, or 1.5 U HotStarTaq DNA polymerase (Qiagen, Hilden, Germany) and 1× PCR buffer (Qiagen). Primer sequences, concentrations and PCR conditions are summarized in Table S5 in Additional file 1.

PCR products were visualized on 1.2% agarose gels, purified using the Gel/PCR DNA Fragments extraction kit (AVEGENE, Taipei, Taiwan) and measured by intercalating fluorescence dye using the Qubit Fluorometer (Qubit HS-Kit, Invitrogen, Darmstadt, Germany). After equimolar amplicon pooling, emulsion PCR was performed using Lib-L emPCR protocols. DNA containing beads were recovered, enriched and sequenced on a XLR70 Titanium PicoTiterPlate (Roche) separated into eight regions, according to the manufacturer's protocols. Reads were extracted from primary sff-files and assigned to the reference sequence. Afterwards, the reads were imported into BiQ Analyzer HT [46] to filter out low quality reads and analyze the methylation levels and patterns. In total, 340 amplicons were sequenced and 331,768 high quality sequences were obtained with an average conversion rate >99%.

### DNA methylation analysis of repetitive elements

DNA methylation levels in the repetitive DNA elements *HERVK *and *LINE1 *(not covered by the Illumina Beadchip) were determined using methylation-dependent primer extension assays (SIRPH). The bisulfite PCRs were performed as described in the previous section (see Table S5 in Additional file 1 for primer sequences, concentrations and PCR conditions). The degree of methylation was determined using single-nucleotide primer extension (SNuPE) assays in combination with ion-pair reversed-phase high-performance liquid chromatography (IP-RP-HPLC) separation techniques (SIRPH) as previously described [47]. In brief, after amplification, unincorporated dNTPs and primers were removed by treating 5 µl PCR product with Exonuclease I/SAP mix (1U each, USB) for 30 minutes at 37°C, followed by an inactivation step of 15 minutes at 80°C. Afterwards, 14 µl primer extension mastermix (2.0 mM MgCl_2_, 0.05 mM ddCTP, 0.05 mM ddTTP, 3.6 µM SNuPE primer, 5 U TERMIPol DNA Polymerase (Solis BioDyne) and 1× reaction buffer C (Solis BioDyne)) were added to the Exonuclease 1/SAP-treated PCR product. A primer extension reaction was performed with a primer annealing next to a CpG (in the context of the original genomic sequence) being extended by either a ddCTP or a ddTTP, depending on whether the site was methylated prior the bisulfite treatment and PCR or not. SNuPE reaction conditions and primer sequences are described in Table S6 in Additional file 1. Obtained SNuPE products were loaded directly on a DNASepTM (Transgenomic, Omaha, USA) column and separated on the WAVE™ system (Transgenomic) using acetonitril gradient elution. The elution gradient parameters were adjusted specifically for each SNuPE primer. MIs were obtained by calculating the ratio AC/(AC+AT), where AC and AT is the area under the peak corresponding to the ddCTP and the ddTTP-extended primer, respectively, as calculated by Wave Maker v4.1 (Transgenomic). Two CpG sites per amplicon were analyzed. The assays were validated using DBS of eight DNA samples (four chorion and four decidua). Correlation coefficients (r) between the SIRPH and the DBS data for CpG_1 _and CpG_2 _of *HERVK *and LINE1 were 0.98, 0.96, 0.73 and 0.82, respectively.

### Whole blood and buccal genome-wide reference methylation data

To generate reference datasets for whole blood and buccal DNA, genome-wide DNA methylation data were obtained from the Gene Expression Omnibus (GEO; Table S1 in Additional file 1). As there are currently no directly comparable 450k datasets available, we used data obtained with the Illumina HumanMethylation27 BeadChip (shares 25,978 CpGs with the 450k chip). Whole blood DNA methylation data were obtained from 274 postmenopausal female controls (GEO accession number [GSE19711]) [48]. Buccal DNA methylation data were obtained from 60 female samples (mean age ± standard deviation 15.1 ± 0.5; GEO accession number [GSE25892]) [49]. Subsequently, genome-wide DNA methylation reference datasets for whole-blood and buccal were generated by averaging the DNA methylation data.

### Data analysis

To determine whether the phenotypic characteristics differed significantly between the heavier and lighter co-twins, a paired *t*-test for continuous data and Fisher's exact test for categorical data were carried out using the statistical package SAS (version 9.2, SAS Institute Inc., Cary, NC, USA). Using the Bioconductor *methylumi *library, the genome-wide methylation data were loaded into R statistical environment for analysis [50]. Quality control of the Infinium methylation data was assessed using the HumMeth27QCReport library. For all different types of methylation data (that is, Infinium, DBS and SIRPH), DNA methylation differences between the heavy and light co-twins were tested using the non-parametric Wilcoxon signed-rank test, which tests the null hypothesis that the mean DNA methylation differences are equal to zero (see Supplemental methods in Additional file 1 for details).

### Power calculation

Power analysis of the Wilcoxon signed rank test is complicated because its power function is difficult to express [51]. Therefore, to estimate the power of our analysis we did the calculation for its closest parametric equivalent (paired *t*-test). With a sample size of 16 discordant twin pairs, 99% power is achieved to detect a mean β-value difference of 0.05 using a two-sided paired *t*-test assuming a standard deviation of 0.025 (which is the true standard deviation observed in our data) and a significance threshold of 0.01. The details of this power calculation and calculations using lower significance thresholds are presented in Table S7 in Additional file 1.

### Accession codes

HumanMethylation450 profiles of the twin samples are available in GEO under accession number [GSE39560]. The DBS data are available in the Sequence Read Archive under accession number [SRA075928].

## Abbreviations

BW-MVP: birth weight-associated methylation variable positions; DBS: deep bisulfite sequencing; DC: dichorionic; DZ: dizygotic; EFPTS: East Flanders Prospective Twin Survey; EWAS: epigenome-wide association study; GEO: Gene Expression Omnibus; HELP: HpaII tiny fragment enrichment by ligation-mediated PCR; IUGR: intra-uterine growth restriction; MC: monochorionic; MI: methylation index; MVP: methylation variable position; MZ: monozygotic; SIRPH: single-nucleotide primer extension assays in combination with ion-pair reversed-phase high-performance liquid chromatography separation techniques; SNP: single nucleotide polymorphism; SNuPE: single nucleotide primer extension; T2D: type 2 diabetes.

## Competing interests

The authors declare that they have no competing interests associated with this manuscript.

## Authors' contributions

NS designed the study, conducted the experimental work, participated in the statistical data analysis and wrote the manuscript. PL performed bioinformatical and statistical analyses and participated in writing the manuscript. ST and JG assisted in the DBS and methylation-dependent primer extension experiments. GG and MR generated the HumanMethylation450 profiles. CD, JF and MZ were involved in the study design, collecting the twin data and samples. JW supervised the study, participated in the study design, writing the manuscript and provided technical and material support. All authors read and approved the final manuscript.

## Supplementary Material

Additional file 1 **Supplemental methods, tables and figures**. Supplemental methods include Infinium HumanMethylation450 data pre-processing, adjustment for cell type heterogeneity, and association analysis and candidate selection. Table S1: DNA methylation profiles used to create the cell-type reference data set. Table S2: cell type-specific quantitative markers used as explanatory variables in heterogeneity adjustment. Table S3: characteristics of the 45 CpG sites that are significantly differentially methylated between the heavy and light co-twins identified using the Infinium HumanMethylation450 BeadChip. Table S4: distribution of the samples across the bead chips, detected CpGs and the corresponding call rate per sample. Table S5: reaction conditions and primer sequences of the bisulfite-PCRs. Table S6: reaction conditions and primer sequences of the SIRPH analysis. Table S7: statistical power of the twin study. Figure S1: pair-wise correlations for each pair of samples, calculated from approximately 480,000 CpGs. Figure S2: sample-independent Infinium methylation controls. Figure S3: sample-dependent Infinium methylation controls. Figure S4: pair-wise correlations for each pair of samples, including the reference dataset for whole-blood and buccal (27k), calculated from approximately 25,978 CpGs. Figure S5: mixing experiment with KG1a and K562 cells profiled on the Infinium HumanMethylation450 BeadChip. Figure S6: distribution of the correlation coefficients of the methylation values of the approximately 480,000 CpGs to the methylation values of the *PTPN7 *CpG (cg18384097). Figure S7: pair-wise correlations for each pair of samples after adjusting for cell type composition using the *PTPN7 *CpG (cg18384097). Figure S8: examples of methylation profiles generated using the deep bisulfite sequencing data of the *APPL2*, *PPARGC1B*, *PHKG2 *and *PTPN7 *amplicons. Figure S9: correlation plots in which the (unadjusted) Infinium 450K data of the validated CpGs are plotted against the (unadjusted) deep bisulfite sequencing (DBS) data for every sample separately. Figure S10: box-plot of the correlation coefficients calculated between the Infinium 450K data and the deep bisulfite sequencing (DBS) data of the validated CpGs for every individual sample. Figure S11: correlation plots of the (unadjusted) Infinium 450K data and the (unadjusted) deep bisulfite sequencing (DBS) data of the 17 discordant MZ twin pairs for each validated CpG separately. Figure S12: continuation of Figure S11. Figure S13: box-plot of the intra-pair differences in β-values of the 64 SNPs present on the Infinium HumanMethylation450 BeadChip before and after normalisation using internal controls and background subtraction by the GenomeStudio software. Click here for file
