# Supplementary material for: Adult monozygotic twins discordant for intra-uterine growth have indistinguishable genome-wide DNA methylation profiles
Source: Genome Biol. 2013 May 26;14(5):R44. doi: 10.1186/gb-2013-14-5-r44 (PMC4054831; doi:10.1186/gb-2013-14-5-r44)
Supplement: Additional file 1 — Supplemental methods, tables and figures. Supplemental methods include Infinium HumanMethylation450 data pre-processing, adjustment for cell type heterogeneity, and association analysis and candidate selection. Table S1: DNA methylation profiles used to create the cell-type reference data set. Table S2: cell type-specific quantitative markers used as explanatory variables in heterogeneity adjustment. Table S3: characteristics of the 45 CpG sites that are significantly differentially methylated between the heavy and light co-twins identified using the Infinium HumanMethylation450 BeadChip. Table S4: distribution of the samples across the bead chips, detected CpGs and the corresponding call rate per sample. Table S5: reaction conditions and primer sequences of the bisulfite-PCRs. Table S6: reaction conditions and primer sequences of the SIRPH analysis. Table S7: statistical power of the twin study. Figure S1: pair-wise correlations for each pair of samples, calculated from approximately 480,000 CpGs. Figure S2: sample-independent Infinium methylation controls. Figure S3: sample-dependent Infinium methylation controls. Figure S4: pair-wise correlations for each pair of samples, including the reference dataset for whole-blood and buccal (27k), calculated from approximately 25,978 CpGs. Figure S5: mixing experiment with KG1a and K562 cells profiled on the Infinium HumanMethylation450 BeadChip. Figure S6: distribution of the correlation coefficients of the methylation values of the approximately 480,000 CpGs to the methylation values of the PTPN7 CpG (cg18384097). Figure S7: pair-wise correlations for each pair of samples after adjusting for cell type composition using the PTPN7 CpG (cg18384097). Figure S8: examples of methylation profiles generated using the deep bisulfite sequencing data of the APPL2, PPARGC1B, PHKG2 and PTPN7 amplicons. Figure S9: correlation plots in which the (unadjusted) Infinium 450K data of the validated CpGs are plotted against the (unadjusted) d [file gb-2013-14-5-r44-S1.DOC]

**Content Additional File 1**

**Supplemental methods**

- Infinium HumanMethylation450 data pre-processing
- Adjustment for cell type heterogeneity
- Association analysis and candidate selection

**Supplemental tables**

- **Table S1.** DNA methylation profiles used to create the cell-type reference data set.
- **Table S2.** Cell type-specific quantitative markers used as explanatory variables in heterogeneity adjustment.
- **Table S3.** Characteristics of the 45 CpG sites that are significantly differentially methylated between the heavy and light co-twins identified using the Infinium HumanMethylation450 BeadChip.
- **Table S4.** Distribution of the samples across the bead chips, detected CpGs and the corresponding call rate per sample.
- **Table S5.** Reaction conditions and primer sequences of the bisulfite-PCRs.
- **Table S6.** Reaction conditions and primer sequences of the SIRPH analysis.
- **Table S7.** Statistical power of the twin study.

**Supplemental figures**

- **Figure S1.** Pair-wise correlations for each pair of samples, calculated from ≈480,000 CpGs.
- **Figure S2.** Sample independent Infinium methylation controls.
- **Figure S3.** Sample dependent Infinium methylation controls.
- **Figure S4.** Pair-wise correlations for each pair of samples, including the reference dataset for whole-blood and buccal (27k), calculated from ≈ 25,978 CpGs.
- **Figure S5.** Mixing experiment with KG1a and K562 cells profiled on the Infinium HumanMethylation450 BeadChip.
- **Figure S6.** Distribution of the correlation coefficients of the methylation values of the ≈480,000 CpGs to the methylation values of the *PTPN7* CpG (cg18384097).
- **Figure S7.** Pair-wise correlations for each pair of samples after adjusting for cell type composition using the *PTPN7* CpG (cg18384097).
- **Figure S8.** Examples of methylation profiles generated using the deep bisulfite sequencing data of the *APPL2*, *PPARGC1B*, *PHKG2* and *PTPN7* amplicons.
- **Figure S9.** Correlation plots in which the (unadjusted) Infinium 450K data of the validated CpGs is plotted against the (unadjusted) deep bisulfite sequencing (DBS) data for every sample separately.
- **Figure S10.** Box-plot of the correlation coefficients calculated between the Infinium 450K data and the deep bisulfite sequencing (DBS) data of the validated CpGs for every individual sample.
- **Figure S11.** Correlation plots of the (unadjusted) Infinium 450K data and the (unadjusted) deep bisulfite sequencing (DBS) data of the 17 discordant MZ twin pairs for each validated CpG separately.
- **Figure S12.** Continuation of Figure S11.
- **Figure S13.** Box-plot of the intra-pair differences in β-values of the 64 SNPs present on the Infinium HumanMethylation450 BeadChip before and after normalisation using internal controls and background subtraction by the GenomeStudio software.

**Supplemental methods**

***Infinium HumanMethylation450 data pre-processing***

The raw data from the Illumina scanner after the internal image processing stage were loaded for initial analysis into Genome Studio software (Illumina, San Diego, CA, USA). The background level – defined as the 5^th^ percentile of the negative control probe signal distribution – was subtracted from the intensities at each probe in each channel separately, setting the negative values to 0. The intensities were then normalized by multiplying the intensity at each probe by the scaling factor. The latter was defined for each sample as the ratio of the mean intensity of (positive) normalization control probes in this sample to same mean intensity in an arbitrary-selected reference sample ("normalization to internal controls"). The data was exported as Genome Studio analysis report, and loaded into R using the *methylumi* library (<http://bioconductor.org/packages/2.5/bioc/html/methylumi.html>). The sample-dependent and sample-independent quality control probes were visualized using the *HumMethQC27* library (see **Figure S2** and **S3**). Low-quality probes were defined as those having a detection *p*-value (one minus quantile of the negative probe intensity distribution into which the intensity of the given probe falls) greater than or equal to 0.001 in one of the samples, and were excluded. The methylation level for each probe was estimated as β-value and M-value [1] using *methylumi* routines. We set the offset α, added to the intensities of methylated and unmethylated probes in order to decrease the influence of the extremely low intensities on the resulting methylation levels, to be equal 25.

***Adjustment for cell type heterogeneity***

Since the uneven cellular composition could undermine the downstream statistical analysis, we decided to normalize the Infinium data so that the methylation level at each probe becomes linearly independent of the cellular composition in the studied sample. In order to do this, we first selected quantitative cell type-specific markers using reference data sets of purified cell types, supposedly present in saliva [2]. We composed the reference data set of publicly available DNA methylation profiles obtained with the Illumina Infinium HumanMethylation27 BeadChip (**Table S1**). The buccal epithelium data was obtained from 60 female samples generated by Essex et al. [3]. Leukocyte data was obtained from Calvanese et al. [4], who generated HumanMethylation27 profiles from purified leukocyte-subtypes (e.g. neutrophils, B-lymphocytes, CD4+ T-lymphocytes, CD8+ T-lymphocytes and natural killers) that were isolated from the same blood pool. Selection of cell type-specific markers was performed on the set of probes, which is shared between the HumanMethylation27 and HumanMethylation450 BeadChip.

Our method is based on the assumption that the methylation level of CpGs that are highly discriminatively methylated between buccal epithelium and leukocytes provide an estimate of the amount of buccal epithelial cell derived DNA present in the saliva samples. As a consequence, such marker CpGs can be used to adjust the methylation data for the varying cell type composition of the saliva samples. We are aware that alternative approaches can be used to estimate the proportion of buccal derived DNA present in the samples (e.g. Principal Component Analysis). However, we prefer to adjust using a “physical” marker (a real CpG). which has the advantage that in the validation phase the marker can be measured using another technology (e.g. deep bisulfite sequencing) and thus also in samples of which no array data (so no PCA estimated cell type proportion) is available.

Although in theory, the relation between the β-value of the marker CpG and the proportion of buccal derived DNA should be linear, in reality, due to technical reasons, the precise behavior of the Infinium methylation data with changing cell type composition can be quite different. This is illustrated by a mixing experiment with KG1a and K562 cells, of which the mixed proportions were profiled on the Infinium HumanMethylation450 BeadChip. In **Figure S5**, the β-values of marker CpGs that were hypermethylated in K562 (and thus hypomethylated in KG1a) are plotted against the corresponding mixing proportions. **Figure S5** indicates that the methylation values of the marker CpGs provide a good estimate of the mixed proportions, but it also shows that many probes do not behave linear. Besides, we observed that marker CpGs often have a different range of variation, e.g. β-value Buccal/ β-value Blood Marker_1 = 0.80/0.20, Marker_2 = 0.90/0.05. Accordingly, an intuitive approach like averaging methylation data of different marker CpGs will not reduce noise and therefore not result in a good cell composition surrogate for the adjustment. Instead we used a model fitting approach to identify that single marker CpG of which the methylation measurements behave the most linear with respect to the changing cell proportions.

The identification of the marker CpGs was performed as follows:

1. For each cell type *c* from the reference data set, all marker CpGs were ranked by the absolute difference between the methylation level in *c* ($M_{c}$) and the average of methylation levels in all other contributing cell types ($\bar{M}_{a}$): $\bar{M}_{a}=\frac{1}{m-1}\sum_{i} M_{i}$ (1)
   where $M_{i}$ is the methylation value of the CpG in the contributing cell type $i$ of all cell types except *c*, and *m* is the number of contributing cell types in the sample.
2. For every considered cell type the top 10 highest ranking CpGs were selected as quantitative marker candidates.
3. The optimal quantitative marker was determined as the one giving the best linear fit to the majority of measured CpGs in the saliva data set. For all the 450k probes 11 linear models were fitted, one single-parameter model for each of the 10 candidate markers and the intercept-only ("zero") model. Then the fit of the 11 models, measured by Akaike information criterion (AIC), was compared and the model with the lowest AIC was considered to give the best linear fit. The candidate marker CpG which was most often generating the best fitting model, was considered to be the most linear quantitative marker.

We realize that the marker CpGs could potentially be affected by genetic differences between the samples of Essex et al. [3] and the pooled sample of Calvanese et al. [4], i.e. due to SNPs in the probes or allele specific methylation (meth-QTLs). In order to exclude this, we carefully examined the methylation levels of the final marker CpGs in the twin data, in which such effects can easily be detected through the small intra-pair and large inter-pair beta-value differences that are observed in case of a SNP or meth-QTL. In addition, we also examined large whole blood DNA methylation (n=274) and buccal DNA methylation (n=60) data sets of Teschendorff et al. [5] and Essex et al. [3], respectively. Moreover, we did an extensive annotation search for potential presence of polymorphisms and/or other genetic variability features in the HumanMethylation450 probe of the marker CpG. The results allowed to be confident that the selected marker CpGs are not affected by genetic variation. In addition, we refer to Figure S12 in which the Infinium 450K data of the *PTPN7* CpG (buccal epithelium marker) is correlated against the deep bisulfite sequencing data. If the variation at this CpG would be genetically driven then the methylation values of the twin pairs would cluster in this figure, which is not the case (except for pair 1, both members of which had extreme high buccal concentrations in their saliva, as revealed by multiple analyses in the study, e.g. array-wide correlation patterns to reference buccal epithelium and blood profiles).

In case the methylation levels of the marker CpGs are correlated (confounded) with the phenotype (low birth weight vs. high birth weight), the adjustment procedure will also remove the DNA methylation changes induced by the intra-uterine growth restriction. However, we verified this and none of the marker CpGs is associated with the phenotype (*p*_paired t-test_>0.10, *p*_Wilcoxon signed rank_>0.10).

Finally, the genome-wide DNA methylation data, expressed as M-values, was adjusted for variation introduced by cellular composition differences using standard linear regression. The selected cell type-specific markers (**Table S2**) were used as explanatory variables in the linear regression model, with the methylation levels measured at each probe as the response variable. First the adjustment was performed using the buccal epithelium marker only. For a given probe the levels were only adjusted if the AIC of the one-parameter model was lower than the AIC of the intercept-only, "zero" model (i.e. CpGs not affected by saliva composition do not get adjusted by this procedure). The residuals of the fitted adjustment model incremented with the intercept term were treated as the adjusted methylation levels. The second adjustment was done using all selected quantitative markers (**Table S2**) as predictors and the best adjustment model for each probe was selected from the set of all possible one- and two-parameter models plus the “zero” model. The adjusted methylation levels were transformed into β-values via the logit-transformation.

***Association analysis and candidate selection.***

The hypothesis that poor prenatal conditions lead to significant DNA methylation differences between the heavy and light co-twins was tested using the non-parametric Wilcoxon signed-rank test on the intra-pair β-value differences obtained for each CpG site independently. The intra-pair difference was defined as β-value observed for the heavier co-twin minus the β-value of the lighter co-twin, and the two-sided null hypothesis was tested for the symmetry of the sample distribution around zero. The calculations were performed using the standard R function *wilcox.test.* First the Wilcoxon signed-rank test was performed on the dataset that was only adjusted for the buccal marker. Subsequently, the analysis was repeated on a dataset that was adjusted for additional blood derived cellular subtypes. CpGs that were significant in both analysis (*p*<0.01) and showed an absolute mean β-value difference >0.05 in both analyses were considered as being stably differentially methylated between the discordant MZ twins. The non-stringent nominal significance threshold of 0.01 was chosen to limit the number of false negatives. To compensate for the substantially increased false positive rate due to multiple testing, an extensive technical validation was carried out.

Since low birth weight is associated with late-onset metabolic diseases, our hypothesis is based on the assumption that the adverse prenatal growth conditions experienced by the smaller co-twins lead to DNA methylation changes that remain throughout life and should therefore be detectable at adult age. Accordingly, the inter-pair age range (22-45 years) of our sample should not affect the results. Nevertheless, it can be that *in utero* induced methylation changes get “diluted” over time. Then the *in utero* induced methylation differences would be stronger in the younger twin pairs compared to the elder ones. Such changes could be uncovered in a stratified analysis, but our study was not designed to identify age dependent effects on *in utero* induced methylation changes and therefore such a stratified analysis on our data is underpowered.

**Supplemental tables**

**Table S1.** DNA methylation profiles used to create the cell-type reference data set.

| Cell type/Tissue | Accession | #samples | Reference | Comments |
| --- | --- | --- | --- | --- |
|  |  |  |  |  |
| *Tissues* |  |  |  |  |
| Whole blood | GEO: [GSE19711] | 274^a^ | [5] |  |
| Buccal epithelium | GEO: [GSE25892] | 66^a^ | [3] | Mouth scrabs |
| *Purified cell types* |  |  |  |  |
| Neutrophils (granulocytes) | GEO: [GSE30090] | 1^b^ | [4] | Centrifugation, negative selection while sorting for other cell types |
| B-lymphocytes | GEO: [GSE30090] | 1^b^ | [4] | Centrifugation, CD19+ staining and sorting |
| CD4+ T-lymphocytes | GEO: [GSE30090] | 1^b^ | [4] | Magnetic bead isolation |
| CD8+ T-lymphocytes | GEO: [GSE30090] | 1^b^ | [4] | Magnetic bead isolation |
| NK cells | GEO: [GSE30090] | 1^b^ | [4] | Centrifugation, CD56+ staining and sorting |
|  |  |  |  |  |

^a^The reference profiles were obtained by averaging the profiles of individual samples. ^b^The reference profiles were obtained by methylation profiling of the pooled samples (5 individuals). GEO = Gene Expression Omnibus.

**Table S2.** Cell type-specific quantitative markers used as explanatory variables in heterogeneity adjustment.

^a^Official Illumina Infinium probe identifier, valid both for Illumina HumanMethylation27 and HumanMethylation450 BeadChips.

| Probe ID^a^ | Cell-type | Buccal epithelium | Neutrophils | B-cells | CD4+ T-cells | CD8+ T-cells | NK-cells | Chr. | Position | Gene |
| --- | --- | --- | --- | --- | --- | --- | --- | --- | --- | --- |
|  |  |  |  |  |  |  |  |  |  |  |
| cg18384097 | Buccal | **0.82** | 0.04 | 0.06 | 0.04 | 0.04 | 0.09 | 1 | 202129566 | *PTPN7* |
| cg20748065 | Neutrophils | 0.79 | **0.02** | 0.78 | 0.92 | 0.85 | 0.69 | 7 | 75583421 | *POR* |
| cg00226923 | B-cells | 0.82 | 0.96 | **0.01** | 0.85 | 0.78 | 0.80 | 6 | 36972027 | *FGD2* |
| cg22858308 | CD4+ T-cells | 0.93 | 0.94 | 0.95 | **0.19** | 0.68 | 0.75 | 6 | 143095613 | *HIVEP2* |
| cg10163825 | CD8+ T-cells | 0.07 | 0.10 | 0.15 | 0.18 | **0.74** | 0.15 | 16 | 776685 | - |
| cg06900776 | NK-cells | 0.28 | 0.02 | 0.01 | 0.10 | 0.12 | **0.78** | X | 100878107 | *ARMCX3* |
|  |  |  |  |  |  |  |  |  |  |  |

**Table S3. Characteristics of the 45 CpG sites that are significantly differentially methylated between the heavy and light co-twins (BW-MVPs) identified using the Infinium HumanMethylation450 BeadChip.**

|  | CpG Number | Mean  β-value difference | | *P*-value | | Chromosomal position | | Gene name | | Gene region | | Relation  to CpG island | | Enhancer | | Affected by buccal | |
| --- | --- | --- | --- | --- | --- | --- | --- | --- | --- | --- | --- | --- | --- | --- | --- | --- | --- |
|  |  | |  | |  | |  | |  | |  | |  | |  | |  |
| 1 | cg16826055 | | 0.06 | | 0.00003 | | Chr 7: 41087207 | |  | |  | |  | | Yes | | Yes |
| 2 | **cg02409150** | | -0.06 | | 0.00003 | | Chr 16: 30764007 | | *PHKG2* | | Body | | S_Shelf | |  | |  |
| 3 | **cg12170649** | | -0.06 | | 0.00006 | | Chr 12: 105622107 | | *APPL2* | | Body | |  | | Yes | |  |
| 4 | **cg26404226** | | -0.05 | | 0.00006 | | Chr 10: 90686467 | |  | |  | |  | | Yes | |  |
| 5 | cg18699337 | | -0.07 | | 0.00009 | | Chr 17: 44159144 | | *KANSL1* | | Body | |  | | Yes | | Yes |
| 6 | cg05518778 | | -0.08 | | 0.00031 | | Chr 7: 148730143 | |  | |  | | S_Shelf | |  | | Yes |
| 7 | cg07574216 | | -0.08 | | 0.00043 | | Chr 3: 156394398 | |  | | 5'UTR, TSS1500 | | S_Shore | |  | | Yes |
| 8 | cg21234955 | | -0.07 | | 0.00043 | | Chr 9: 97713896 | | *C9orf3* | | Body | |  | | Yes | | Yes |
| 9 | cg12149795 | | -0.05 | | 0.00043 | | Chr 21: 47882121 | | *DIP2A* | | Body | | S_Shelf | |  | | Yes |
| 10 | cg14696311 | | -0.07 | | 0.00058 | | Chr 13: 114855198 | | *RASA3* | | Body | | S_Shelf | | Yes | | Yes |
| 11 | cg09683440 | | 0.06 | | 0.00058 | | Chr 16: 83869927 | |  | |  | |  | | Yes | | Yes |
| 12 | cg02350090 | | -0.06 | | 0.00058 | | Chr 5: 1951736 | |  | |  | | S_Shore | |  | |  |
| 13 | **cg14123607** | | 0.07 | | 0.00076 | | Chr 9: 72164709 | | *APBA1* | | 5'UTR | |  | |  | |  |
| 14 | cg05680237 | | 0.06 | | 0.00101 | | Chr 6: 27103185 | |  | |  | | N_Shelf | |  | | Yes |
| 15 | cg13071869 | | -0.06 | | 0.00101 | | Chr 3: 112948716 | | *BOC* | | 5'UTR | |  | | Yes | | Yes |
| 16 | cg03839714 | | -0.07 | | 0.00131 | | Chr 10: 31361534 | |  | |  | |  | | Yes | | Yes |
| 17 | cg22979546 | | -0.05 | | 0.00131 | | Chr 15: 28389947 | | *HERC2* | | Body | |  | |  | | Yes |
| 18 | **cg15049370** | | -0.07 | | 0.00168 | | Chr 5: 149186389 | | *PPARGC1B* | | Body | |  | | Yes | | Yes |
| 19 | cg04416414 | | 0.07 | | 0.00168 | | Chr 19: 14260587 | | *LPHN1* | | 3'UTR | | N_Shore | | Yes | |  |
| 20 | cg10984962 | | -0.07 | | 0.00168 | | Chr 2: 236462202 | | *AGAP1* | | Body | |  | | Yes | | Yes |
| 21 | cg14868128 | | -0.06 | | 0.00168 | | Chr 6: 22367352 | |  | |  | |  | | Yes | | Yes |
| 22 | cg08846459 | | -0.06 | | 0.00168 | | Chr 5: 2176047 | |  | |  | |  | |  | |  |
| 23 | **cg15487251** | | -0.05 | | 0.00168 | | Chr 3: 185544216 | | *IGF2BP2* | | TSS1500 | | S_Shore | |  | | Yes |
| 24 | cg25064052 | | -0.05 | | 0.00168 | | Chr 4: 166216151 | | *KLHL2* | | Body | |  | | Yes | | Yes |
| 25 | cg21450228 | | -0.05 | | 0.00214 | | Chr 3: 23727711 | |  | |  | |  | | Yes | | Yes |
| 26 | cg10773972 | | -0.07 | | 0.00269 | | Chr 15: 81035670 | | *FAM108C1* | | Body | |  | | Yes | | Yes |
| 27 | cg20438460 | | -0.05 | | 0.00269 | | Chr 2: 146580994 | |  | |  | |  | | Yes | | Yes |
| 28 | cg15940337 | | -0.05 | | 0.00269 | | Chr 8: 142456489 | | *FLJ43860* | | Body | | S_Shelf | | Yes | | Yes |
| 29 | cg08561071 | | 0.06 | | 0.00336 | | Chr 19: 41627236 | | *CYP2F1* | | Body | | N_Shelf | |  | | Yes |
| 30 | cg10156499 | | -0.05 | | 0.00336 | | Chr 11: 112161291 | |  | |  | | S_Shore | | Yes | | Yes |
|  |  | |  | |  | |  | |  | |  | |  | |  | |  |
|  | CpG Number | | Mean  β-value difference | | *P*-value | | Chromosomal position | | Gene name | | Gene region | | Relation  to CpG island | | Enhancer | | Affected by buccal |
|  |  | |  | |  | |  | |  | |  | |  | |  | |  |
| 31 | cg02832477 | | -0.06 | | 0.00418 | | Chr 2: 121501895 | |  | |  | | S_Shelf | |  | |  |
| 32 | cg13181022 | | 0.05 | | 0.00418 | | Chr 12: 69247976 | | *CPM* | | 3'UTR | |  | |  | |  |
| 33 | cg00587523 | | 0.06 | | 0.00516 | | Chr 17: 46212998 | | *SKAP1* | | 3'UTR | |  | |  | |  |
| 34 | cg24994002 | | 0.06 | | 0.00516 | | Chr 5: 14118611 | |  | |  | |  | | Yes | |  |
| 35 | cg04416247 | | -0.05 | | 0.00516 | | Chr 3: 85213671 | | *CADM2* | | Body | |  | | Yes | | Yes |
| 36 | cg24049629 | | 0.07 | | 0.00763 | | Chr 3: 50376475 | | *RASSF1* | | TSS1500, Body | | N_Shore | |  | | Yes |
| 37 | cg25828093 | | 0.07 | | 0.00763 | | Chr 8: 26041003 | |  | |  | |  | | Yes | | Yes |
| 38 | **cg22768222** | | 0.06 | | 0.00763 | | Chr 6: 45383690 | | *RUNX2* | | Body | | N_Shelf | |  | |  |
| 39 | cg12394706 | | 0.06 | | 0.00763 | | Chr 8: 99988676 | |  | |  | | S_Shore | |  | | Yes |
| 40 | **cg10362113** | | 0.06 | | 0.00763 | | Chr 14: 96978537 | | *PAPOLA* | | Body | |  | | Yes | | Yes |
| 41 | cg20540235 | | -0.08 | | 0.00919 | | Chr 8: 91683243 | |  | |  | |  | | Yes | | Yes |
| 42 | cg13035743 | | 0.06 | | 0.00919 | | Chr 6: 32119685 | | *PRRT1* | | 1^st^ Exon, 5'UTR | | S_Shore | | Yes | | Yes |
| 43 | cg26544458 | | -0.06 | | 0.00919 | | Chr 12: 109045006 | | *CORO1C* | | Body | |  | | Yes | |  |
| 44 | cg12222588 | | 0.05 | | 0.00919 | | Chr 18: 34823808 | | *CELF4* | | 3'UTR | | Island | |  | | Yes |
| 45 | cg07965300 | | 0.05 | | 0.00919 | | Chr 12: 56368138 | | *RAB5B* | | 5'UTR | | Island | |  | | Yes |
|  |  | |  | |  | |  | |  | |  | |  | |  | |  |

**Bold and underlined** CpG sites were validated using deep bisulfite sequencing. β-value difference = β-value heavy co-twin – β-value light co-twin, Chr = chromosome, NA = not applicable, Shelves = 2-4 kb from CpG island, Shores = 0-2 kb from CpG island, TSS200 = within 200 bp from transcription start site, TSS1500 = within 1500 bases from transcription start site, UTR = untranslated region.

**Table S4.** Distribution of the samples across the beadchips, detected CpGs (detection *p*-value<0.001) and the corresponding call rate per sample.

| Pair | Twin | Array | Row | Column | Detected CpGs^a^ | Call rate^b^ |
| --- | --- | --- | --- | --- | --- | --- |
|  |  |  |  |  |  |  |
| 1 | H | 1 | 1 | 1 | 481967 | 99.9 |
|  | L | 1 | 2 | 1 | 481792 | 99.9 |
|  |  |  |  |  |  |  |
| 2 | H | 1 | 3 | 1 | 481942 | 99.9 |
|  | L | 1 | 4 | 1 | 481839 | 99.9 |
|  |  |  |  |  |  |  |
| 3 | H | 1 | 5 | 1 | 481902 | 99.9 |
|  | L | 1 | 6 | 1 | 481826 | 99.9 |
|  |  |  |  |  |  |  |
| 4 | H | 1 | 1 | 2 | 481937 | 99.9 |
|  | L | 1 | 2 | 2 | 481923 | 99.9 |
|  |  |  |  |  |  |  |
| 5 | H | 1 | 3 | 2 | 481876 | 99.9 |
|  | L | 1 | 4 | 2 | 481968 | 99.9 |
|  |  |  |  |  |  |  |
| 6 | H | 1 | 5 | 2 | 480062 | 99.5 |
|  | L | 1 | 6 | 2 | 481727 | 99.8 |
|  |  |  |  |  |  |  |
| 7 | H | 2 | 1 | 1 | 482018 | 99.9 |
|  | L | 2 | 2 | 1 | 481996 | 99.9 |
|  |  |  |  |  |  |  |
| 8 | H | 2 | 3 | 1 | 481949 | 99.9 |
|  | L | 2 | 4 | 1 | 481913 | 99.9 |
|  |  |  |  |  |  |  |
| 9 | H | 2 | 5 | 1 | 481880 | 99.9 |
|  | L | 2 | 6 | 1 | 481048 | 99.7 |
|  |  |  |  |  |  |  |
| 10 | H | 2 | 1 | 2 | 481644 | 99.8 |
|  | L | 2 | 2 | 2 | 481941 | 99.9 |
|  |  |  |  |  |  |  |
| 11 | H | 2 | 3 | 2 | 481934 | 99.9 |
|  | L | 2 | 4 | 2 | 481941 | 99.9 |
|  |  |  |  |  |  |  |
| 12 | L | 2 | 5 | 2 | 481800 | 99.9 |
|  | H | 2 | 6 | 2 | 481775 | 99.9 |
|  |  |  |  |  |  |  |
| 13 | H | 3 | 2 | 2 | 481873 | 99.9 |
|  | L | 3 | 3 | 2 | 481843 | 99.9 |
|  |  |  |  |  |  |  |
| 14 | H | 3 | 4 | 2 | 481300 | 99.8 |
|  | L | 3 | 5 | 2 | 481767 | 99.9 |
|  |  |  |  |  |  |  |
| 15 | L | 3 | 6 | 2 | 481663 | 99.8 |
|  | H | 4 | 1 | 1 | 481806 | 99.9 |
|  |  |  |  |  |  |  |
| 16 | H | 4 | 2 | 1 | 481839 | 99.9 |
|  | L | 4 | 3 | 1 | 481845 | 99.9 |
|  |  |  |  |  |  |  |
| 17 | L | 5 | 5 | 2 | 481888 | 99.9 |
|  | H | 5 | 6 | 2 | 481772 | 99.9 |
|  |  |  |  |  |  |  |
|  |  |  |  |  |  |  |

^a^Number of CpGs with a detection *p*-value<0.001. ^b^Call rate (%), based on a detection *p*-value <0.001. Total number of CpGs are 482421. H = high birth weight, L = low birth weight.

**Table S5.** Reaction conditions and primer sequences of the bisulfite-PCRs.

| Method | CpG number | Gene/  element | Forward primer sequence (5’→3’) | C | Reverse primer sequence (5’→3’) | C | T | Cyc | Product size | #CpGs |
| --- | --- | --- | --- | --- | --- | --- | --- | --- | --- | --- |
|  |  |  |  |  |  |  |  |  |  |  |
| DBS^a^ | cg14123607 | *APBA1* | ATGATATGGTTTATGAATTTTAATTTTT | 83 | AAAAAATTATATCTCCTCTATCCCAATA | 83 | 55 | 45 | 306 bp | 9 |
|  | cg12170649 | *APPL2* | TATTGAATGTGGTATAGGTTAAGTATTT | 83 | AATTAAACATCCCAAATTTAAAAA | 83 | 58 | 45^c^ | 228 bp | 4 |
|  | cg26404226 | Chr10q23.3 | GTTTATGGAAAGAATGATTTTTGTTT | 83 | CCCTACCAAAACCTAAATCTCAA | 83 | 56 | 45^c^ | 228 bp | 2 |
|  | cg15487251 | *IGF2BP2* | TTAGTTTTAAAGTTAGGGTGGTGG | 83 | AATTTCATTTCCTAACTAAAACCAAA | 83 | 54 | 42^c^ | 280 bp | 14 |
|  |  | *HNF4A* | GGGAAGTTATTGAATTAGGGGATT | 83 | ACCCTCTCTACCTTCCTTTCAAAC | 83 | 59 | 42^c^ | 394 bp | 9 |
|  | cg10362113 | *PAPOLA* | TTATTATAGGGGGTTATTTTAGTTTTT | 83 | CAAACTAATCTAAACTCTTAAACTCAAA | 83 | 55 | 42^c^ | 308 bp | 3 |
|  | cg02409150 | *PHKG2* | GAGAGTGTTTAGTAGTGGATTGATTAT | 83 | TCCTCAAAATAACATTATCAAATACTA | 83 | 53 | 45^b^ | 369 bp | 6 |
|  | cg15049370 | *PPARGC1B* | TTTTAGATGGAGTGTGTGGGATAT | 83 | CACAATAACTCAAACCTATAATTCCAA | 83 | 62 | 45^c^ | 339 bp | 11 |
|  | cg18384097 | *PTPN7* | TAAGAGGGTATTTTTAGGAGGGAGT | 83 | AAAATATTAAATCACCAAAACAACAAAA | 83 | 54 | 40^c^ | 253 bp | 6 |
|  | cg22768222 | *RUNX2* | AATTTTTTTTTTTTGTAATGTTTTTTT | 83 | CCATCTAATCATAATCAATTTCTAAAAA | 83 | 53 | 45^b^ | 312 bp | 4 |
|  |  |  |  |  |  |  |  |  |  |  |
| SIRPH |  | *HERVK* | TATTTTTTAATTTTAAGTATTTAGGGAT | 200 | ATACCTTCCTCTTATCTCAACTACA | 200 | 55 | 30 | 237 bp | 6 |
|  |  | LINE1^d^ | TTATTAGGGAGTGTTAGATAGTGGG | 200 | CCTCTAAACCAAATATAAAATATAATCT | 200 | 55 | 30 | 246 bp | 18 |
|  |  |  |  |  |  |  |  |  |  |  |

^a^Amplicons were generated using region-specific primers having on their 5´-ends the recommended GS-FLX A and B adaptors (Lib-L) that included multiplex identifiers (MID). ^b^HotStarTaq DNA polymerase (Qiagen) was used. ^c^2 µg Hotstart-IT Binding Protein (USB, Cleveland, USA) added. ^d^Primers from [6]. Bisulfite-treated DNA was denatured for 15 min at 95°C, followed by *n* cycles of 1 min at 95°C, 1 min at T°C and 1 min at 72°C, and a final extension step of 5 min at 72°C. C = concentration (nM), Cyc = number of cycles, DBS = deep bisulfite sequencing, LTR = long terminal repeat, SIRPH = single-nucleotide primer extension (SNuPE) assays in combination with ion pair reverse phase high performance liquid chromatography (IP-RP-HPLC) separation techniques, T = annealing temperature (°C), #CpGs = number of CpGs present in the amplicon.

**Table S6.**Reaction conditions and primer sequences of the SIRPH analysis.

| Gene/element | Region | SNuPE primer 1 | SNuPE primer 2 | Acetonitril Gradient | Oven Temperature |
| --- | --- | --- | --- | --- | --- |
|  |  |  |  |  |  |
| *HERVK* | LTR | 5’-TAGGGATATAAAAATTG-3’ | 5’-GGAAAGATTTGAT-3’ | 15 min: 5%-7.5% | 50°C |
| LINE1^a^ | 5’ UTR CpG island | 5’-CCTAACTCCTTAC-3’ | 5’-CCCCTTTCTTTAACTC-3’ | 13 min: 4.75%-7.25% | 50°C |
|  |  |  |  |  |  |

SNuPE reactions were performed starting with 2 min denaturation at 96°C followed by 50 cycles of 96°C for 20 s, 50°C for 30 sec and 60°C for 30 sec. Products are loaded directly onto the DNASepTM column (Transgenomic) and separated applying the respective acetonitril gradient. ^a^Extended with ddATP and ddGTP. LTR = long terminal repeat.

**Table S7.** Statistical power of the twin study.

| Magnitude of the correlation | Power at alpha = 0.01 | Power at alpha = 1*10^-4^ | Power at alpha = 1*10^-6^ |
| --- | --- | --- | --- |
|  |  |  |  |
| 0.0 | 0.99 | 0.65 | 0.11 |
| 0.2 | 1.00 | 0.80 | 0.21 |
| 0.4 | 1.00 | 0.94 | 0.40 |
| 0.6 | 1.00 | 1.00 | 0.75 |
| 0.8 | 1.00 | 1.00 | 1.00 |
| 1.0 | 1.00 | 1.00 | 1.00 |
|  |  |  |  |

The table shows the statistical power to detect a mean β-value difference of (at least) 0.05 with a sample size of 16 twin pairs based on a standard deviation of 0.025 (which is the true median standard deviation observed in the data) using a two-sided paired T-test.

**Supplemental figures**

**
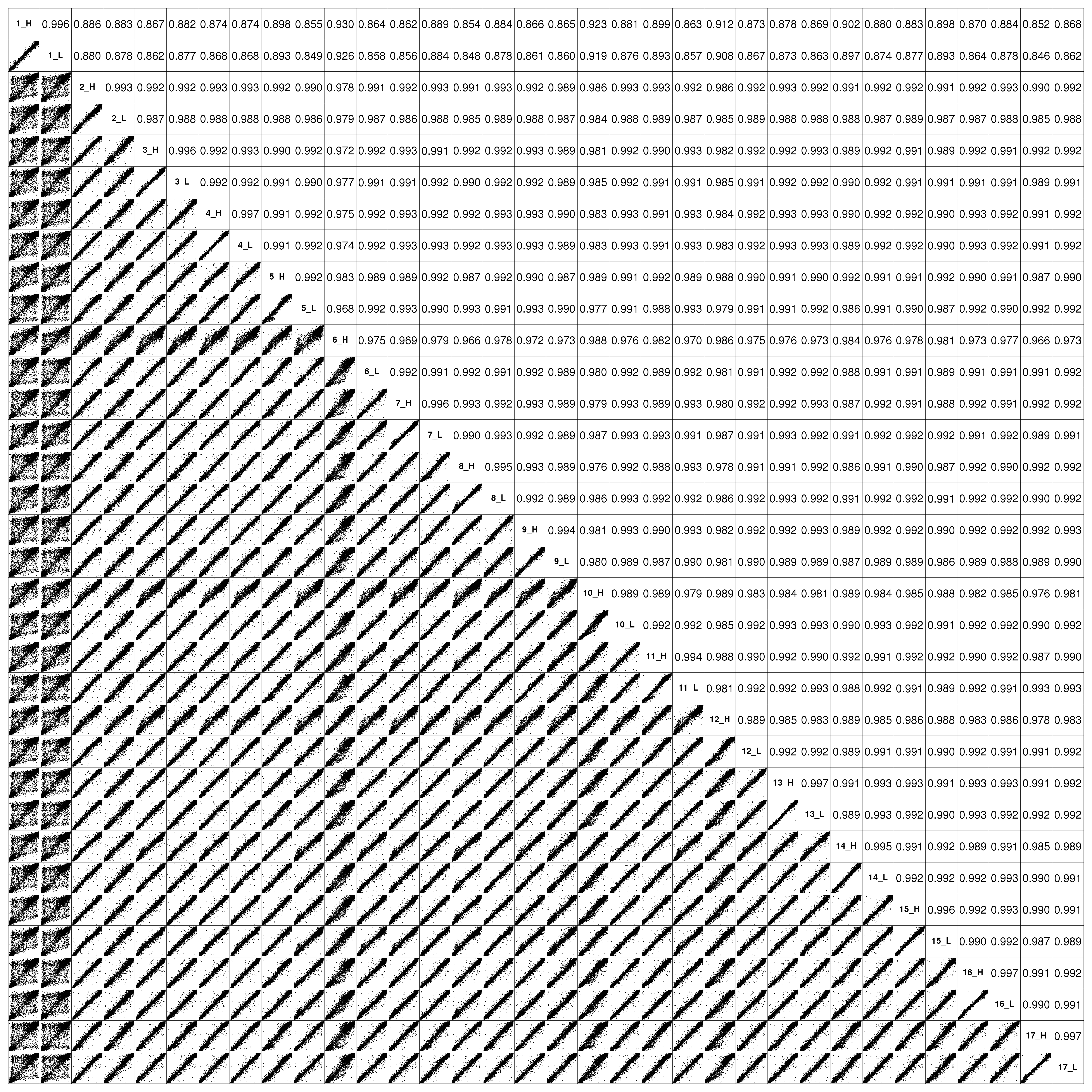
**

**Figure S1.** Pair-wise correlations for each pair of samples, calculated from ≈480,000 CpGs. Sample labels are shown on the diagonal. Pearson correlation coefficients are shown in the upper part of the figure and the dotplots under the diagonal illustrate a visual representation of the similarity between two samples (H = high birth weight, L = low birth weight).

**
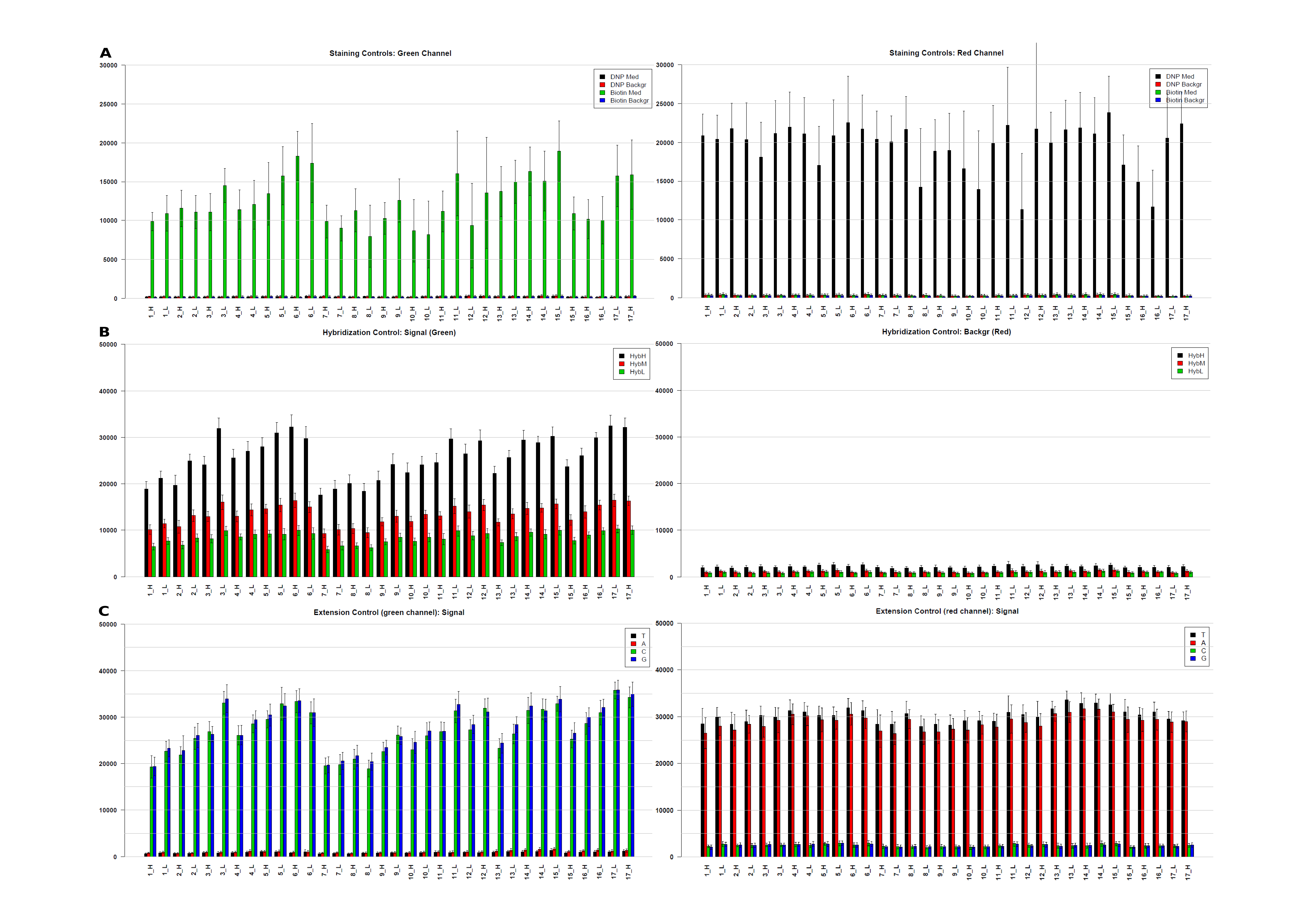
**


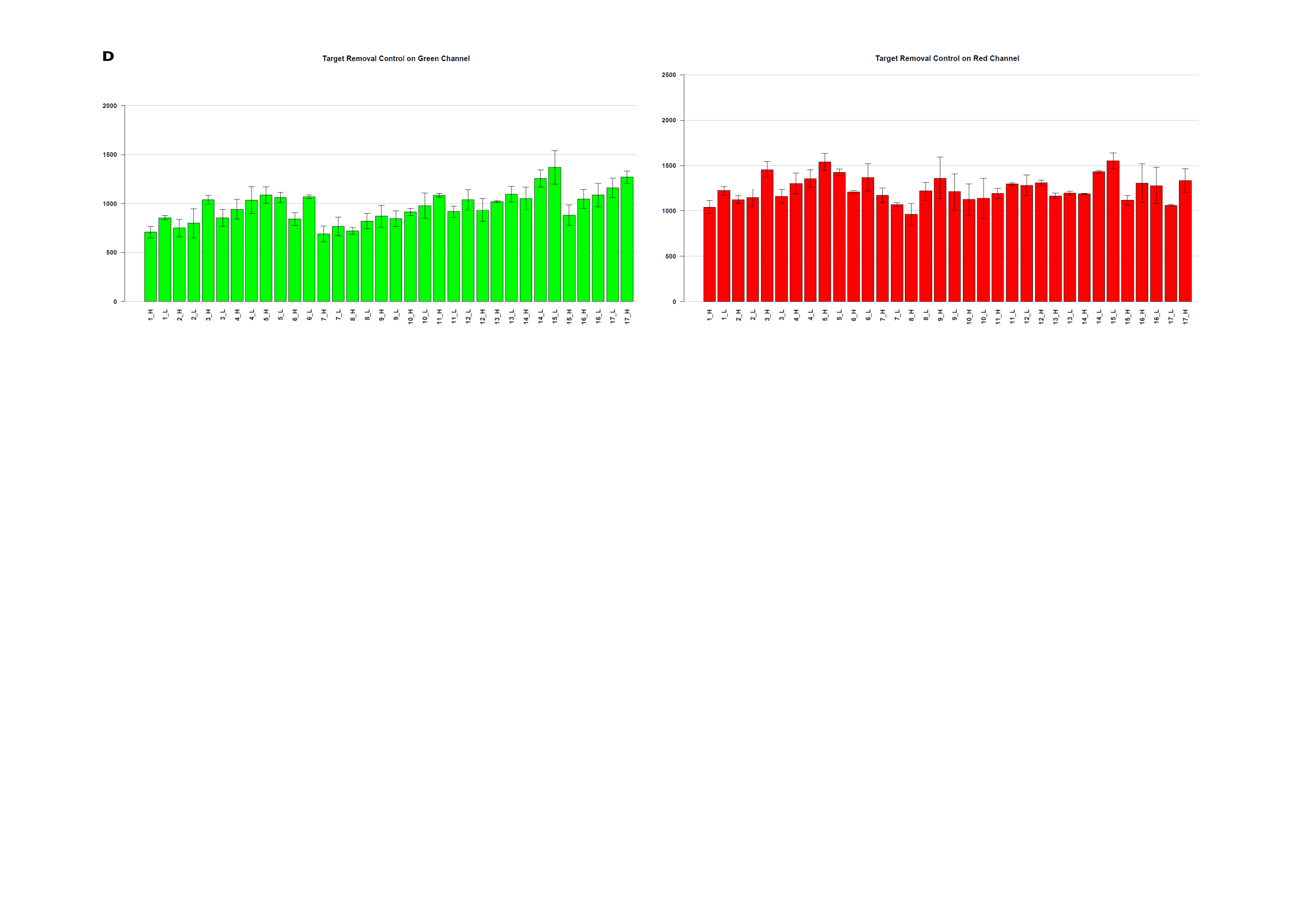
**Figure S2. Sample independent Infinium methylation controls.** **A) Staining controls** are used to examine the efficiency of the staining step in both the red and green channels, and are independent of the hybridization and extension step. **B) Hybridization controls** test the overall performance of the Infinium assay using synthetic targets that are present in the hybridization buffer at three levels (high (5 pM), medium (1 pM) and low concentration (0.2 pM)) and complement the sequence on the array perfectly, which allows the probe to extend on the synthetic target as a template. The performance of the hybridization controls should be monitored only in the green channel. **C) Extension controls** test the extension efficiency of A, T, C, and G nucleotides from a hairpin probe, and their performance should be monitored in the red (A,T) and green (C,G) channels. **D) Target removal controls** test the efficiency of the stripping step after the extension reaction. Target removal controls are present in the hybridization buffer RA1 and these oligos are extended using the probe sequence as a template. This process generates labelled targets and extension from the probe does not occur. All target removal controls should result in low signal compared to the hybridization controls, indicating that the targets were removed efficiently after extension. H = high birth weight, L = low birth weight.


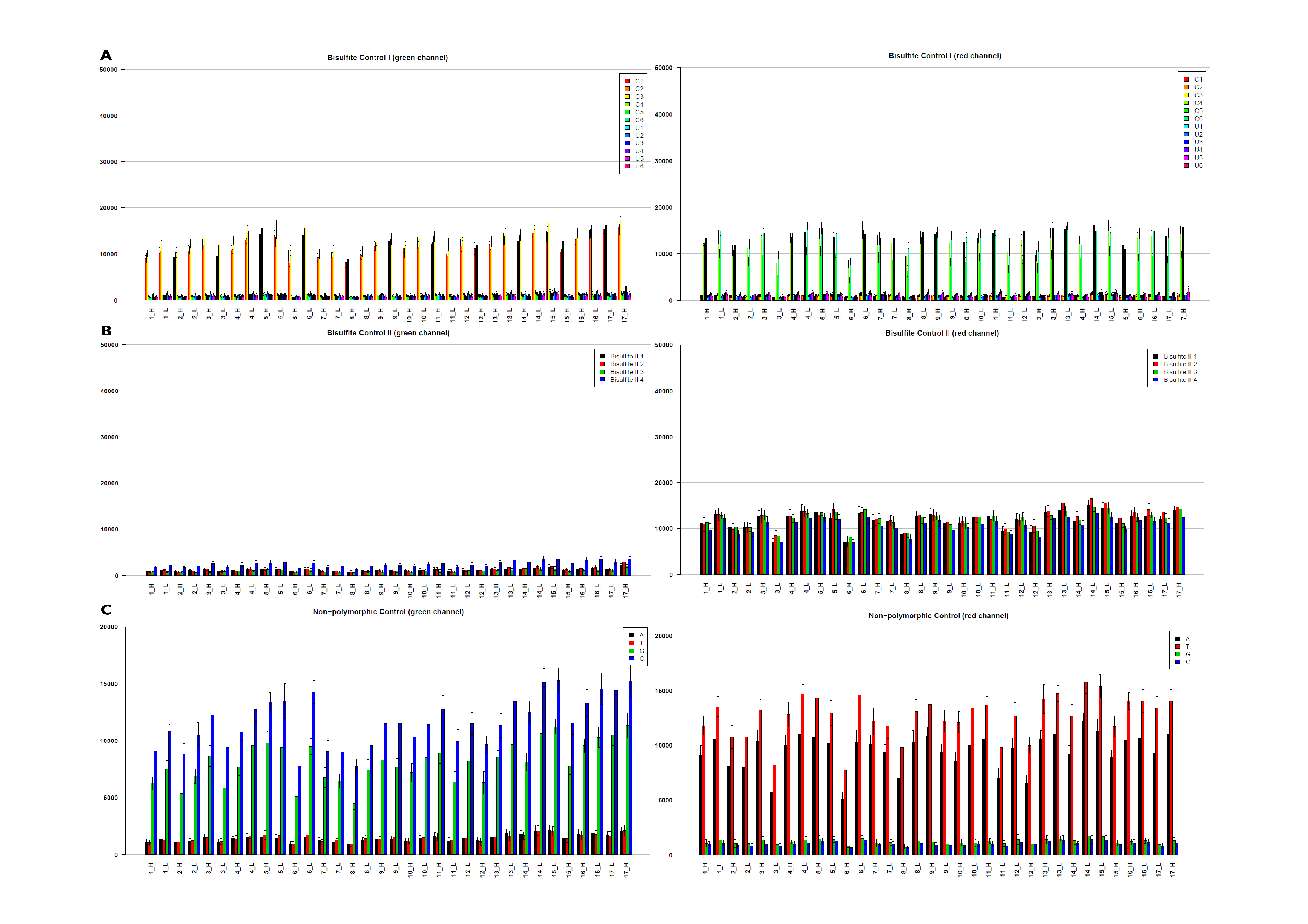


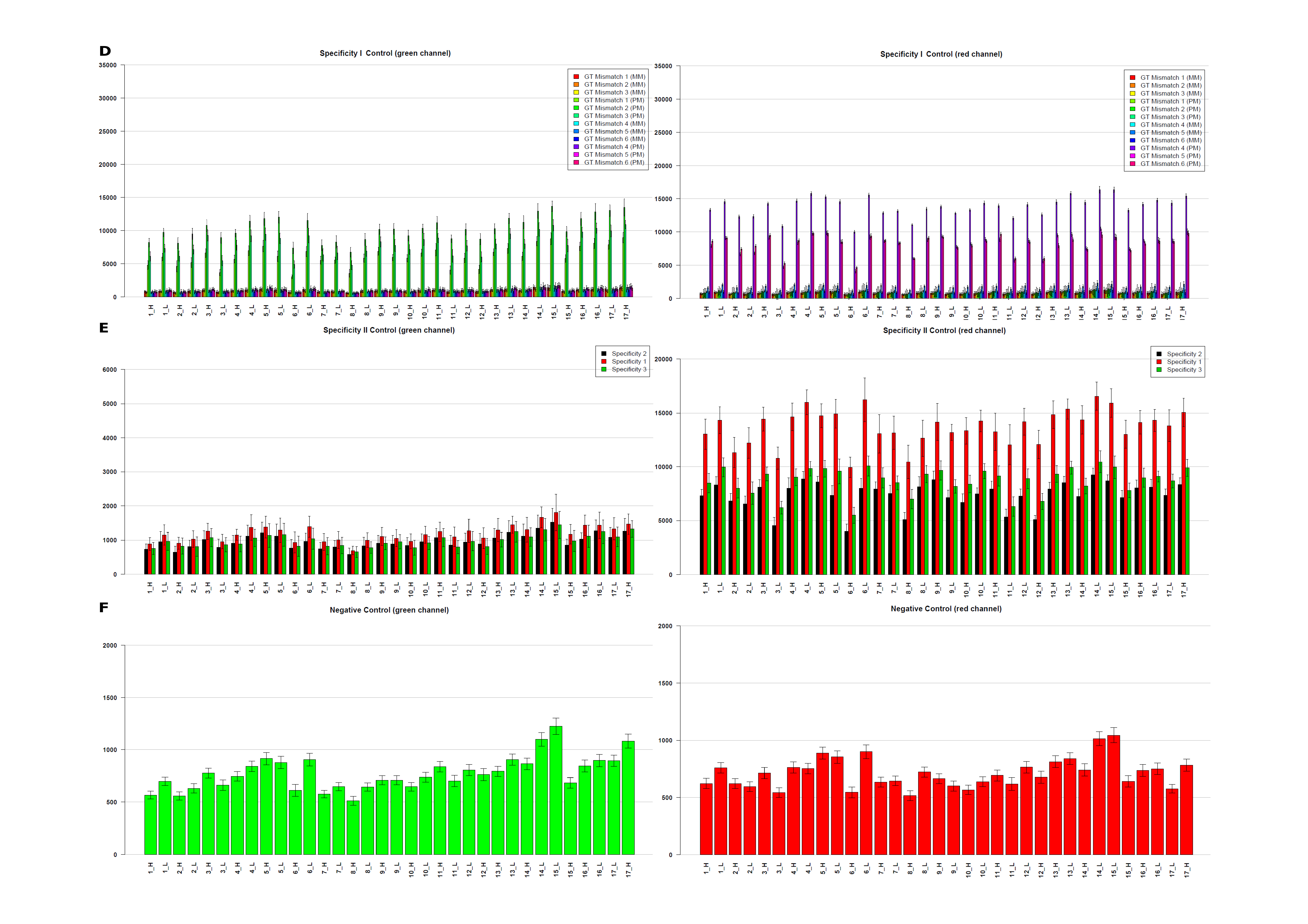


**Figure S3. Sample dependent Infinium methylation controls.** **A)** **Bisulfite controls I** use the Infinium I probe design to monitor the efficiency of the bisulfite conversion. If the bisulfite conversion was successful, the "C" (converted) probes will match the converted sequence and get extended. If the sample has unconverted DNA, the "U" (unconverted) probes will get extended. Performance of bisulfite controls C1, C2 and C3 should be monitored in the green channel, and controls C4, C5 and C6 should be monitored in red channel. **B) Bisulfite controls II** use the Infinium II probe design to monitor efficiency of bisulfite conversion. If the bisulfite conversion reaction was successful, the "A" base will get incorporated and the probe will have intensity in the red channel. If the sample has unconverted DNA, the "G" base will get incorporated across the unconverted cytosine, and the probe will have an elevated signal in the green channel. **C) Non-polymorphic controls** test the overall performance of the assay, from amplification to detection, by querying a particular base in a non-polymorphic region of the genome. They allow comparing assay performance across different samples. One non-polymorphic control has been designed for each of the four nucleotides (A, T, C, and G). **D) Specificity I controls** are designed to monitor extension specificity for the Infinium I probes. G/T mismatch controls check for non-specific detection of methylation signal over unmethylated background. PM controls correspond to A/T perfect match and should give a high signal. MM controls correspond to G/T mismatch and should give a low signal. Performance of GT mismatch controls should be monitored in both green and red channels. **E) Specificity II controls** are designed to monitor extension specificity for the Infinium II probes. Specificity II probes should incorporate the "A" base across the non-polymorphic T and have intensity in the red channel. In case of nonspecific incorporation of the "G" base, the probe will have elevated signal in the green channel. **F) Negative controls** target bisulfite-converted sequences that do not contain CpG dinucleotides. Assay probes are randomly permutated and should not hybridize to the DNA template. The mean signal of these probes defines the system background. The performance of the negative controls should be monitored in both the green and red channel. H = high birth weight, L = low birth weight.


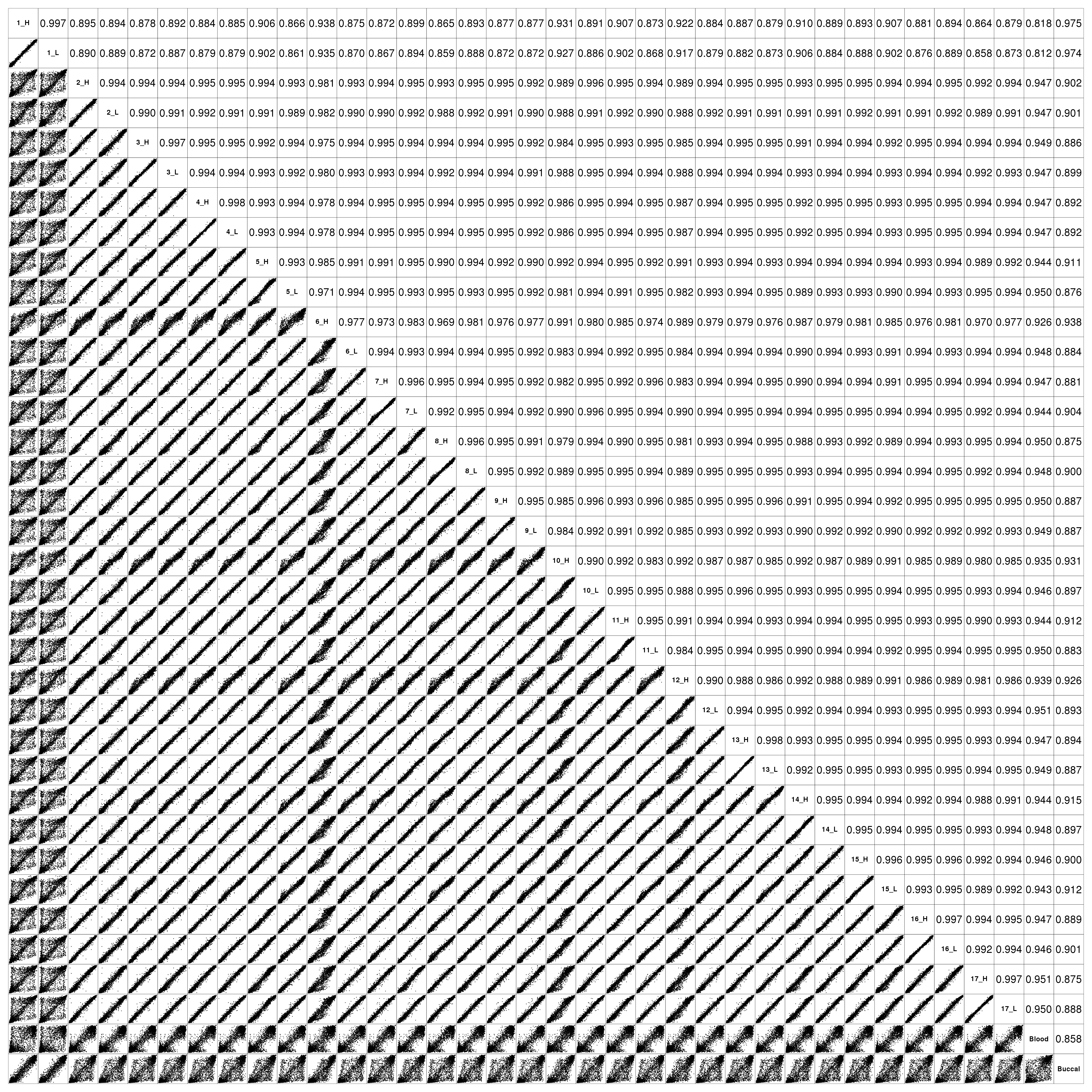
**Figure S4.** Pair-wise correlations for each pair of samples, including the reference dataset for whole-blood and buccal (27k), calculated from ≈25,978 CpGs. Sample labels are shown on the diagonal. Pearson correlation coefficients are shown in the upper part of the figure and the dotplots under the diagonal illustrate a visual representation of the similarity between two samples. H = high birth weight, L = low birth weight.

**
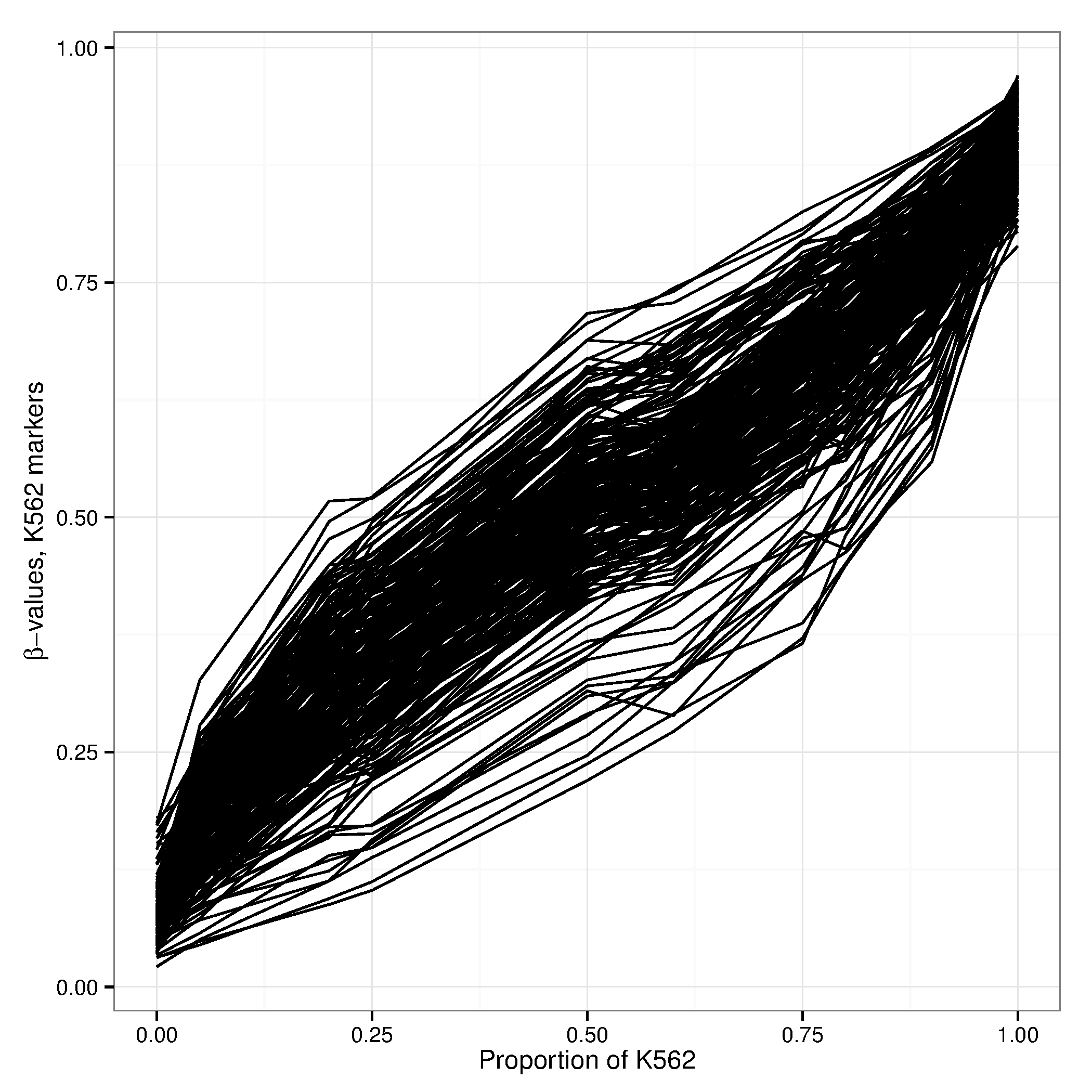
Figure S5.** Mixing experiment with KG1a and K562 cells profiled on the Infinium HumanMethylation450 BeadChip. β-values of marker CpGs hypermethylated in K562 cells (and thus hypomethylated in KG1a cells) plotted against the corresponding mixing proportions.

**
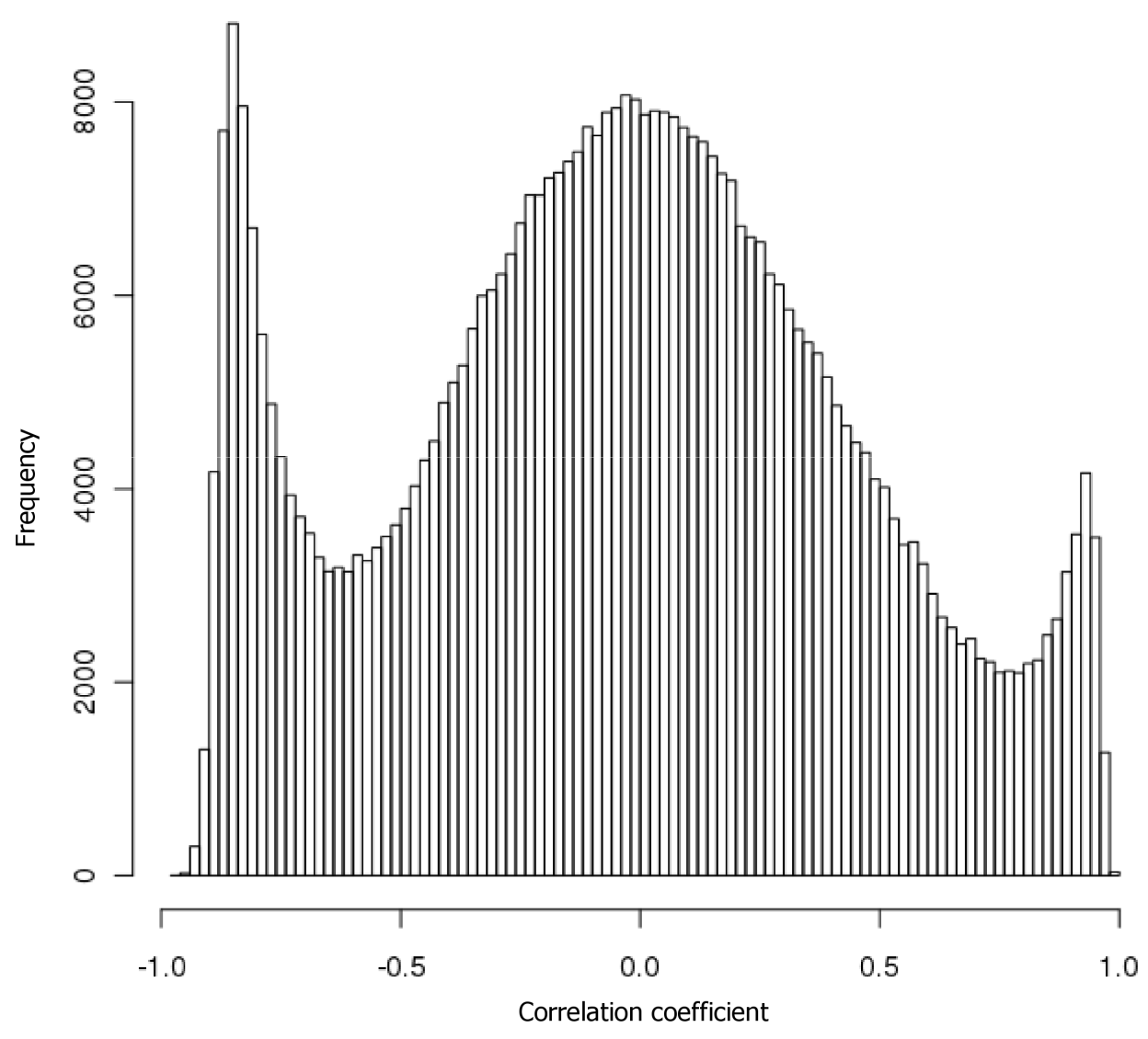
**

**Figure S6.** Distribution of the correlation coefficients of the methylation values of the ≈480,000 CpGs to the methylation values of the *PTPN7* CpG (cg18384097).

**
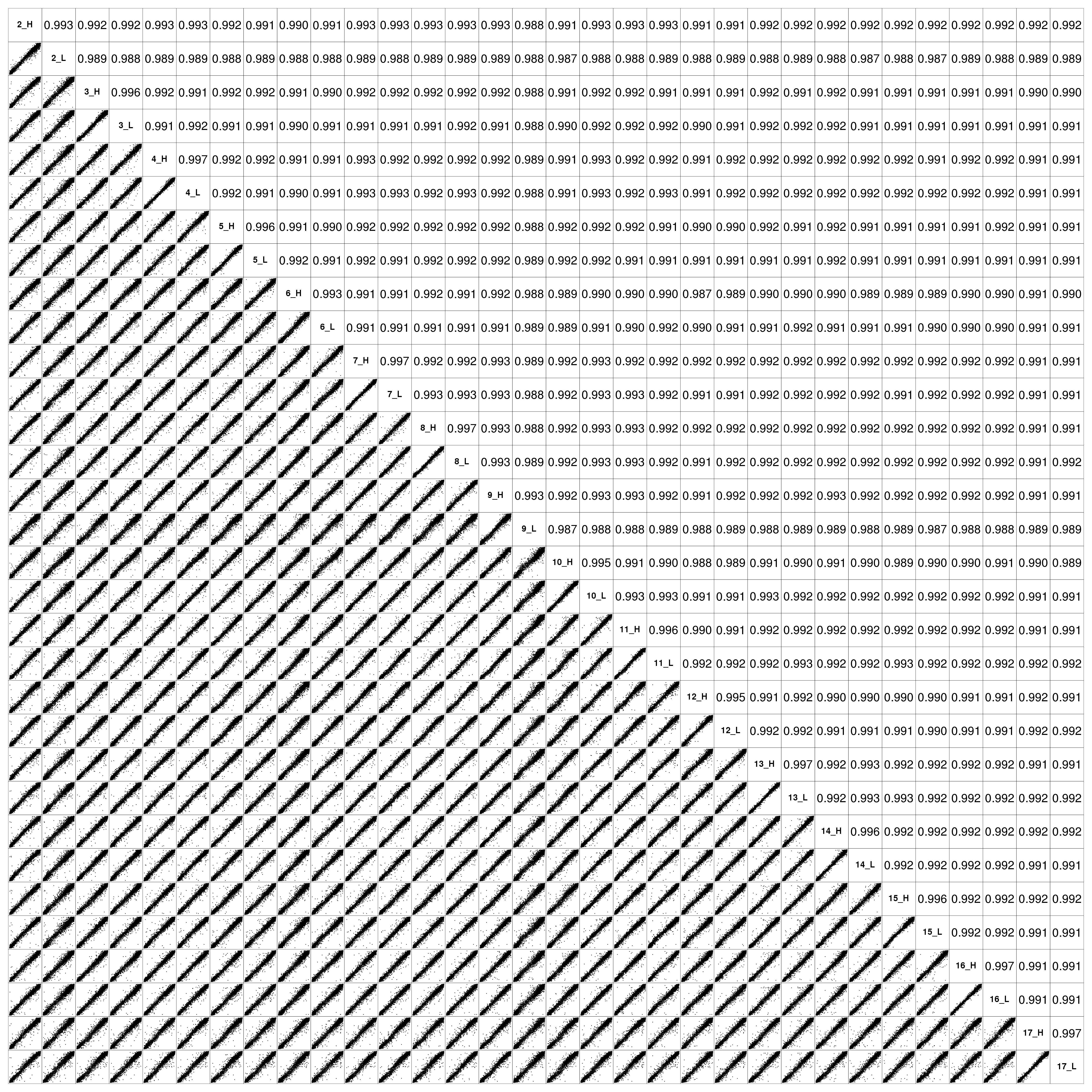
Figure S7.** Pair-wise correlations for each pair of samples after adjusting for cell type composition using the *PTPN7* CpG (cg18384097), calculated from ≈480,000 CpGs. Sample labels are shown on the diagonal. Pearson correlation coefficients are shown in the upper part of the figure and the dotplots under the diagonal illustrate a visual representation of the similarity between two samples. H = high birth weight, L = low birth weight.


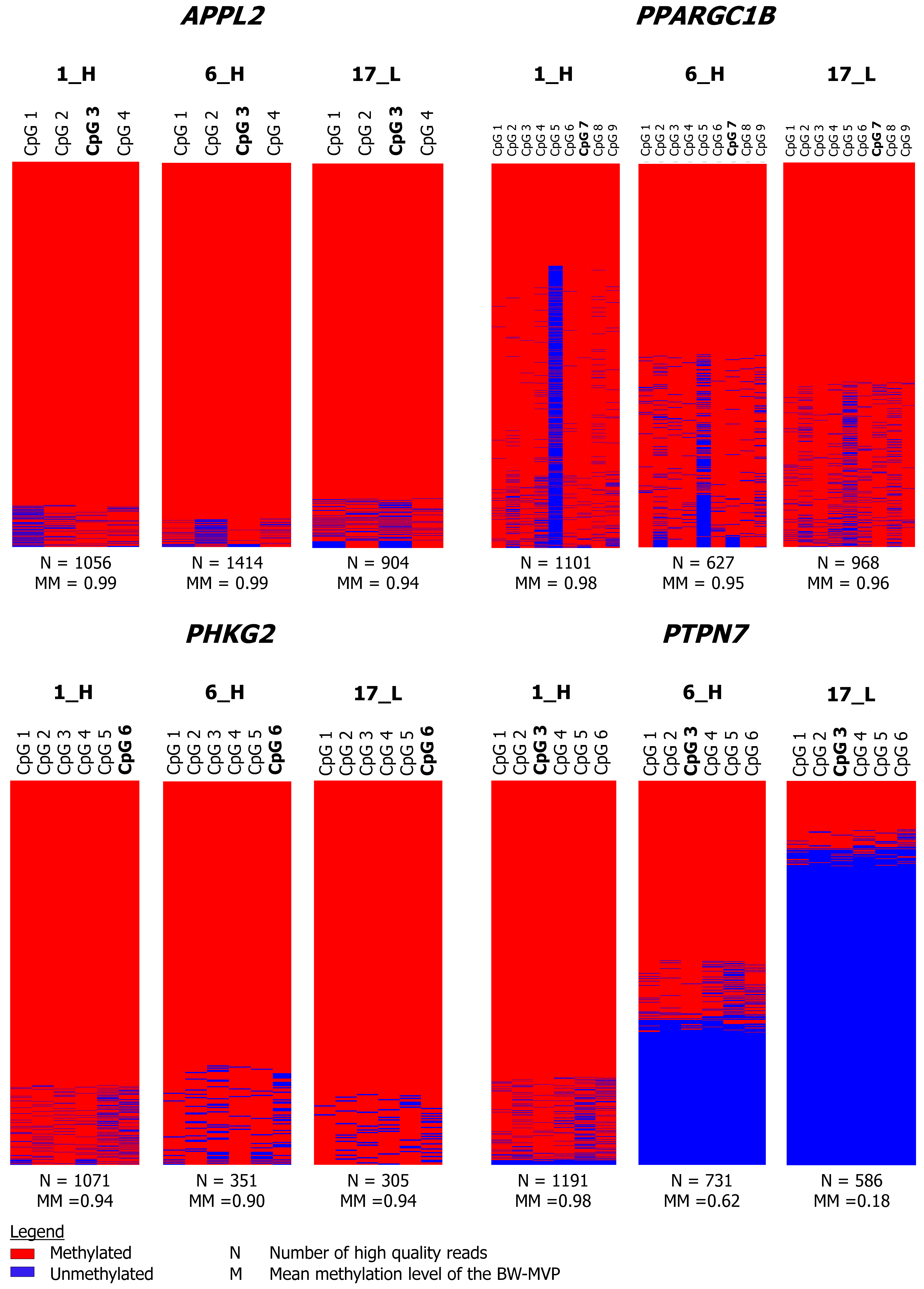


**Figure S8.** Examples of methylation profiles generated using the deep bisulfite sequencing data of the *APPL2*, *PPARGC1B*, *PHKG2* and *PTPN7* amplicons. The **bold CpGs** correspond to the BW-MVPs identified using the Infinium HumanMethylation450 BeadChip.


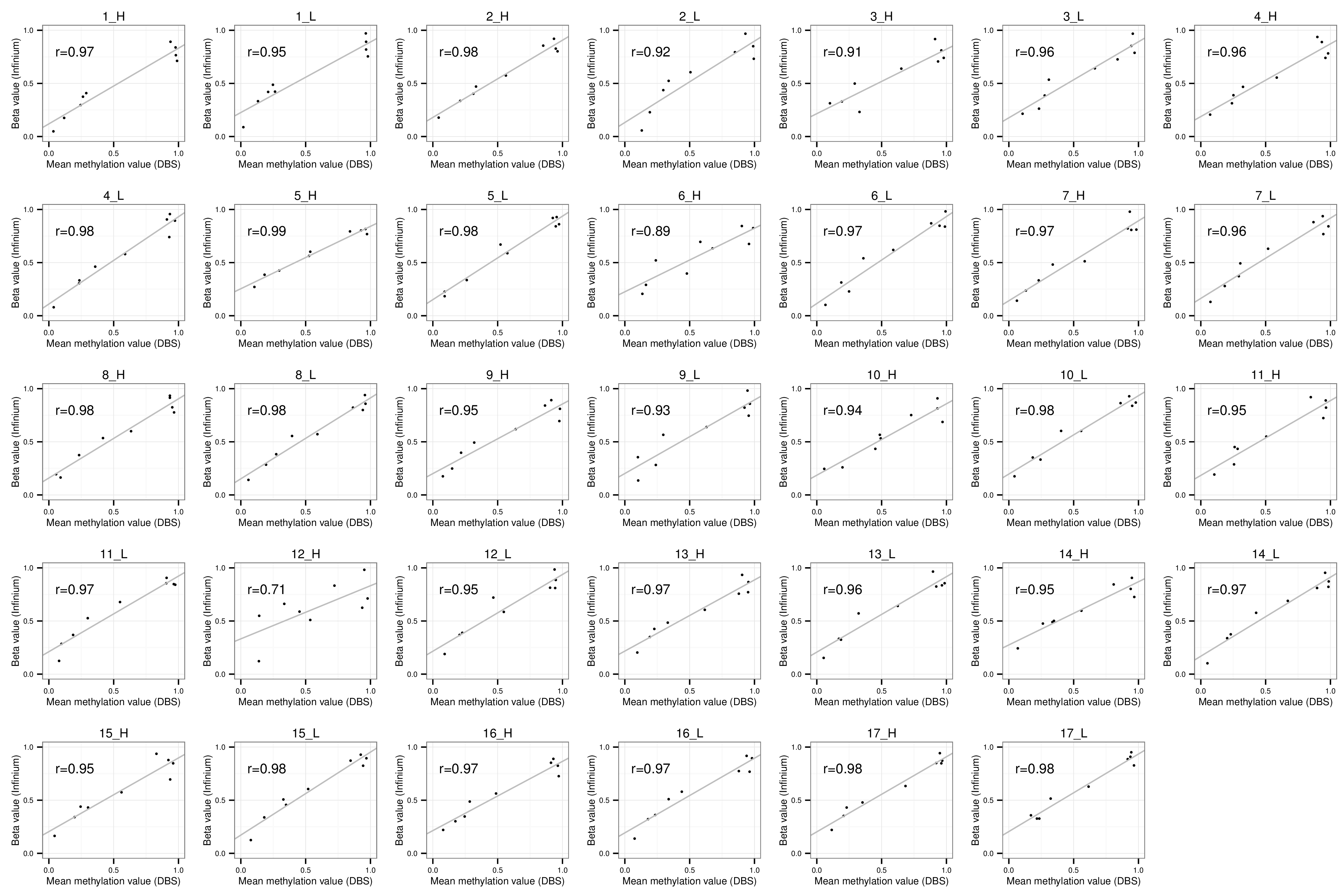
**Figure S9.** Correlation plots in which the (unadjusted) Infinium 450K data of the validated CpGs is plotted against the (unadjusted) deep bisulfite sequencing (DBS) data for every sample separately. Infinium data are expressed as β-value. DBS data are expressed as mean methylation level, in which the methylation level is calculated by dividing the number of reads in which the particular CpG is methylated by the total number of sequenced reads. H = high birth weight, L = low birth weight.


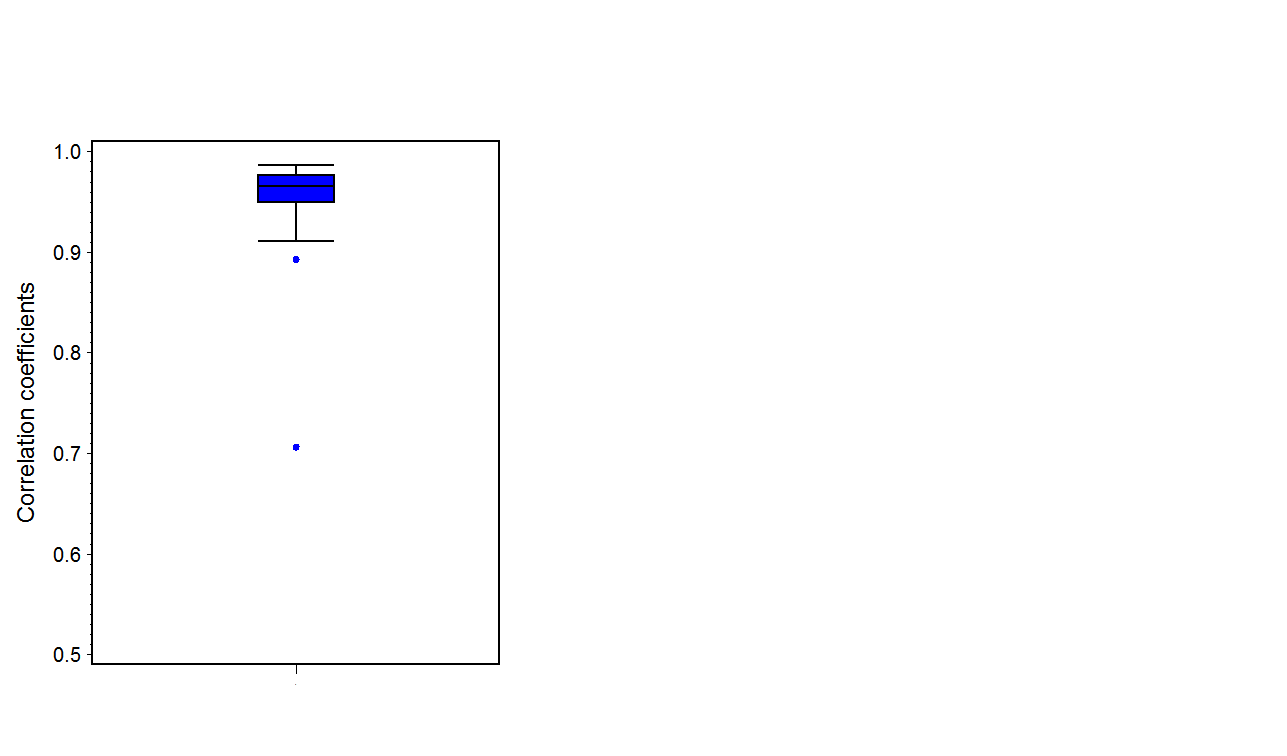
**Figure S10.** Box-plot of the correlation coefficients calculated between the Infinium 450K data and the deep bisulfite sequencing (DBS) data of the validated CpGs for every individual sample.

**
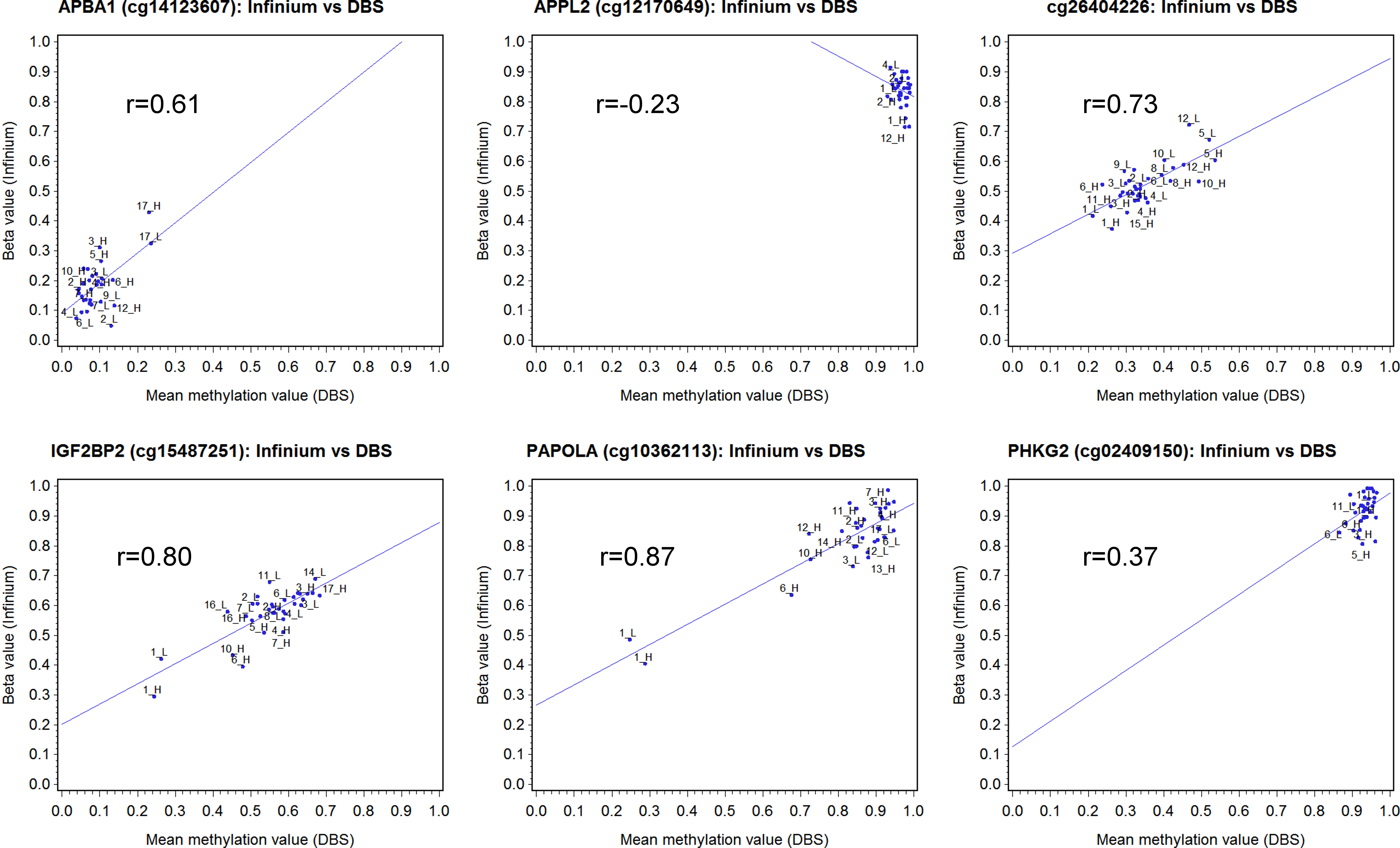
Figure S11.** Correlation plots of the (unadjusted) Infinium 450K data and the (unadjusted) deep bisulfite sequencing (DBS) data of the 17 discordant MZ twin pairs for each validated CpG separately. Infinium data are expressed as β-value. DBS data are expressed as mean methylation level, where the methylation level is calculated by dividing the number of reads in which the particular CpG is methylated by the total number of sequenced reads. H = high birth weight, L = low birth weight.

**
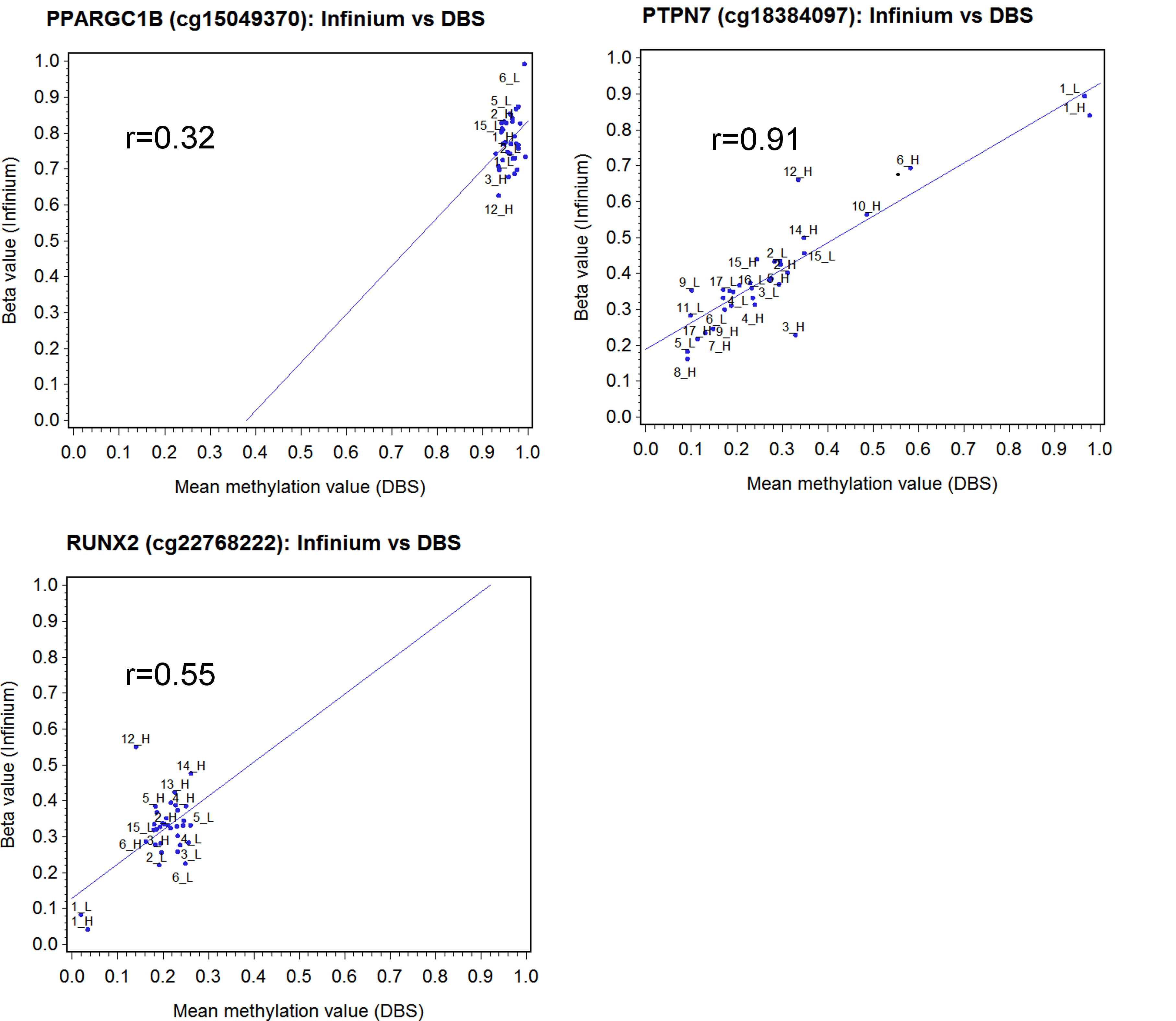
Figure S12.** Continuation of Figure S11.


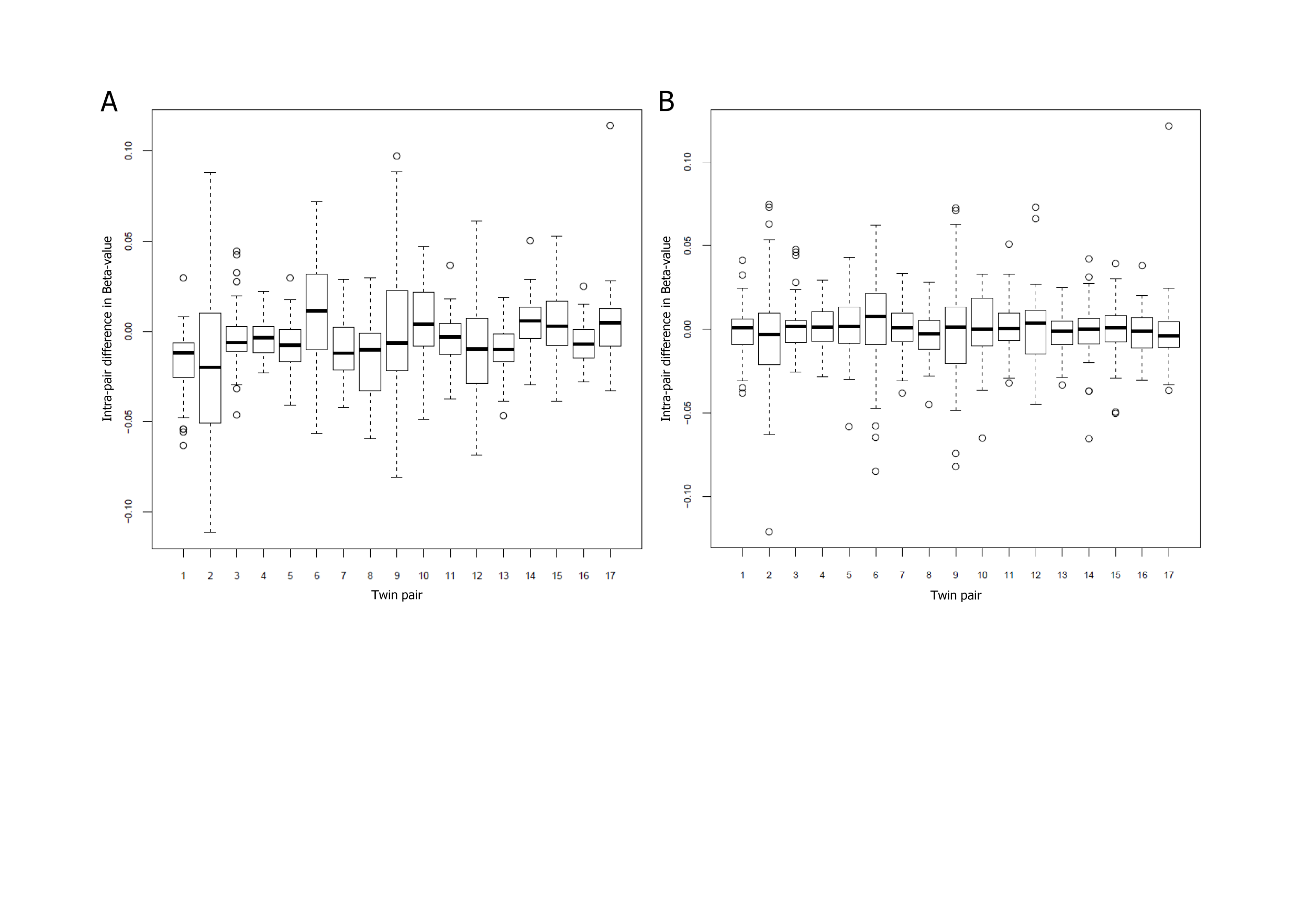
**Figure S13.** Box-plot of the intra-pair differences in Beta-values of the 64 SNPs present on the Infinium HumanMethylation450 BeadChip before (A) and after (B) normalisation using internal controls and background subtraction by the GenomeStudio software (Methylation Module v1.8).

**References**

1. Du P, Zhang X, Huang CC, Jafari N, Kibbe WA, Hou L, Lin SM: **Comparison of Beta-value and M-value methods for quantifying methylation levels by microarray analysis**. *BMC Bioinformatics* 2010, **11**:587.

2. Vidovic A, Vidovic Juras D, Vucicevic Boras V, Lukac J, Grubisic-Ilic M, Rak D, Sabioncello A: **Determination of leucocyte subsets in human saliva by flow cytometry**. *Arch Oral Biol* 2012, **57**:577-583.

3. Essex MJ, Thomas Boyce W, Hertzman C, Lam LL, Armstrong JM, Neumann SM, Kobor MS: **Epigenetic vestiges of early developmental adversity: childhood stress exposure and DNA methylation in adolescence**. *Child Dev* 2013, **84**:58-75.

4. Calvanese V, Fernandez AF, Urdinguio RG, Suarez-Alvarez B, Mangas C, Perez-Garcia V, Bueno C, Montes R, Ramos-Mejia V, Martinez-Camblor P, Ferrero C, Assenov Y, Bock C, Menendez P, Carrera AC, Lopez-Larrea C, Fraga MF: **A promoter DNA demethylation landscape of human hematopoietic differentiation**. *Nucleic Acids Res* 2012, **40**:116-131.

5. Teschendorff AE, Menon U, Gentry-Maharaj A, Ramus SJ, Weisenberger DJ, Shen H, Campan M, Noushmehr H, Bell CG, Maxwell AP, Savage DA, Mueller-Holzner E, Marth C, Kocjan G, Gayther SA, Jones A, Beck S, Wagner W, Laird PW, Jacobs IJ, Widschwendter M: **Age-dependent DNA methylation of genes that are suppressed in stem cells is a hallmark of cancer**. *Genome Res* 2010, **20**:440-446.

6. Marques CJ, Costa P, Vaz B, Carvalho F, Fernandes S, Barros A, Sousa M: **Abnormal methylation of imprinted genes in human sperm is associated with oligozoospermia**. *Mol Hum Reprod* 2008, **14**:67-74.
